# Steering the Self‐Assembly Outcome of a Single NDI Monomer into Three Morphologically Distinct Supramolecular Assemblies, with Concomitant Change in Supramolecular Polymerization Mechanism

**DOI:** 10.1002/advs.201900577

**Published:** 2019-06-14

**Authors:** Grzegorz Markiewicz, Maarten M. J. Smulders, Artur R. Stefankiewicz

**Affiliations:** ^1^ Faculty of Chemistry Adam Mickiewicz University Uniwersytetu Poznan´skiego 8 61‐614 Poznan´ Poland; ^2^ Center for Advanced Technologies Adam Mickiewicz University Uniwersytetu Poznan´skiego 10 61‐614 Poznan´ Poland; ^3^ Laboratory of Organic Chemistry Wageningen University Stippeneng 4 6708 WE Wageningen The Netherlands

**Keywords:** naphthalene diimides, noncovalent self‐assembly, nonequilibrium assemblies, self‐assembly mechanism, supramolecular polymers

## Abstract

Noncovalent self‐assembly creates an effective route to highly sophisticated supramolecular polymers with tunable properties. However, the outcome of this assembly process is highly dependent on external conditions. In this work, a monomeric naphthalene diimide (NDI), designed to allow solubility in a wide range of solvents, can assemble into three distinct noncovalent supramolecular species depending on solvent composition and temperature. Namely, while the self‐assembly in chlorinated solvents yields relatively short, hydrogen‐bonded nanotubes, the reduction of solvent polarity changes the assembly outcome, yielding π–π stacking polymers, which can further bundle into a more complex aggregate. The obtained polymers differ not only in their global morphology but—more strikingly—also in the thermodynamics and kinetics of their supramolecular self‐assembly, involving isodesmic or two‐stage cooperative assembly with kinetic hysteresis, respectively. Ultimately, three distinct assembly states can be accessed in a single experiment.

## 1 Introduction

Noncovalent self‐assembly of supramolecular systems^1^ has gained significant interest over the past 30 years as a simple, yet powerful, approach for the synthesis of biomimetic systems^2^ with novel functionalities and high complexity.^3^ Although the artificial assemblies are still behind their biological archetypes in terms of sophistication and functionality,^4^ an impressive amount of work has been done to develop and, more importantly, to understand the complexity of those systems. That is, in the last 5–10 years, understanding has increased far beyond simply monitoring the spontaneous (i.e., thermodynamically controlled) assembly of a single monomer into a supramolecular quasi‐1D polymer.^5^

Instead, research has shifted toward more sophisticated nonequilibrium systems^6^ where competing pathways allow formation of several (kinetic) assembly products,[qv: 5a,7] some of which can be formed under living polymerization conditions.[qv: 2a,8] For example, Fukui et al.[qv: 8b] presented an elegant approach to the living polymerization of porphyrin‐based H‐ and J‐type assemblies, whose assembly into metastable 1D or 2D aggregates could be steered by molecular design of the self‐assembling porphyrin monomer. The Würthner group^9^ has reported that kinetic off‐pathway H‐aggregates of a perylene bisimide dye (formed upon rapid cooling) can be transformed into the thermodynamically favored J‐aggregates by the addition of seeds of the latter type of aggregates. Recently, Cai et al.^10^ reported that bridged naphthalene diimide (NDI) oligomers display dual‐path self‐assembly; upon assembly, H‐ and J‐type aggregates are formed sequentially in solution. Detailed spectroscopic studies, supported by modeling, could even reveal that the H‐ and J‐type aggregates display cooperative and anticooperative self‐assembly mechanisms, respectively.

Moreover, more recently scientists have harnessed multiple noncovalent interactions,[qv: 3d,11] operative under different conditions, as a means to prepare multidimensional supramolecular polymers.[qv: 2b,8b,11a,12] These systems are of particular interests, as the interplay between those interactions may yield polymers which, like in biological systems,[qv: 2a] may be formed outside of thermodynamic equilibrium,^6^ and thus their properties might be tuned by very mild factors.[qv: 5a,7a,c,13] For example, the Hermans group^14^ has shown that, by the control over electrostatic and *π‐π* stacking interactions, it is possible to obtain dissipative supramolecular assemblies from peptide‐functionalized perylenediimides.

Overall, scientists are more and more appreciating that the outcome of a supramolecular polymerization reaction can be highly dependent on the external conditions, resulting in certain cases in pathway complexity, or in the possibility of different reaction outcomes (i.e., type of assembly that is formed). In addition, external parameters that were traditionally considered to be innocuous, like solvent^15^ or salts,^16^ have been found to have dramatic effects on supramolecular polymerization processes. For example, the Aida group^17^ presented the thermally bisignate supramolecular polymerization of porphyrins, where the combination of temperature and the nature of the alcoholic solvent, capable of competing with H‐bond formation in the supramolecular assembly, triggered a change in the assembly pathway. In extreme cases, even trace contaminants in the solvent can drastically affect the self‐assembly process, as was recently reported by the Meijer group,^18^ who showed that traces of water in hydrocarbon solvents play a crucial role during self‐assembly, introducing an additional force, i.e., the hydrophobic effect to the system, which, till date, was largely overlooked for extremely nonpolar media.^19^

Here we show that it is possible to change from a thermodynamically controlled to a kinetically controlled noncovalent supramolecular polymerization, and that the outcome of the assembly is dependent on the solvent composition. In contrast to most supramolecular systems, whose assembly is driven by one major type of noncovalent interaction,[qv: 5a,13e] our monomer building block, an amino acid–derived NDI monomer, was designed with two dominant noncovalent motifs of comparable strength, allowing further external control over assembly outcome and polymerization mechanism by choice of solvent and temperature. Through the proper design of the monomer—in terms of the relative strength of the incorporated noncovalent *π–π*, hydrogen bond, and van der Waals interactions—we were able to obtain not one or two, but three, distinctly different supramolecular morphologies. In each of the three supramolecular architectures generated, the importance and strength of the intermolecular interactions that create them are varying, which in turn leads to a controlled change in their morphology. Furthermore, by choosing the appropriate assembly conditions, we were able to access three supramolecular states in a controlled and reversible manner, acquiring invaluable information on the kinetics and thermodynamics of each of them. In all, we report the synthesis and characterization of a complex noncovalent supramolecular system with three morphologically and thermodynamically distinct noncovalent assemblies, generated from a single molecule.

**Figure 1:**
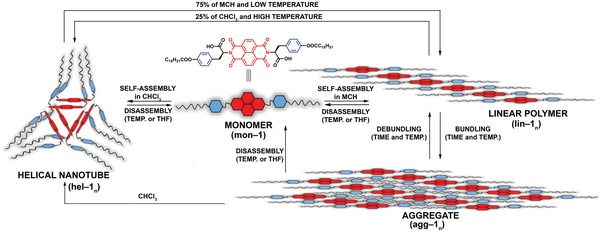
The schematic representation of the self‐assembly and disassembly of **1** in CHCl_3_ and MCH solutions upon changes in temperature and solvent polarity.

Although the self‐assembly of amino acid–derived NDIs has been investigated in the past, especially by the Sanders and Pantoş research groups,^20^ we present that these compounds still conceal their sophisticated assembly routes to—and between—distinct supramolecular structures. Namely, we present the self‐assembly of a newly synthesized tyrosine‐derived NDI monomer, in which several noncovalent interaction sites, i.e., multiple H‐bonding, *π‐π* stacking, and van der Waals forces, were implemented in a single molecule. The precise control over the assembly conditions allowed us to selectively activate/inhibit the particular intermolecular interactions, triggering the self‐assembly from the monomers into three different supramolecular aggregates, with different thermodynamic and kinetic stabilities, and also enabled us to steer the outcome of those assemblies by external stimuli (**Figure** **1**).

## 2 Results and Discussion

The target monomer NDI‐[l‐Tyr(OCOC_15_H_31_)COOH]_2_ (**1**) was synthesized using a three‐step procedure, starting from commercially available 1,4,5,8‐naphthalenetetracarboxylic acid dianhydride, *t*‐butyl protected l‐tyrosine, and palmitoyl chloride, in accordance with a previously described procedure for a C_4_ acylated derivative (see the Supporting Information for details).^12^ Acylation of the tyrosine hydroxyl groups with palmitoyl substituents not only considerably increased the solubility of **1** in almost all organic solvents, ranging from dimethyl sulfoxide (DMSO), through ethers, chlorinated solvents up to hydrocarbons, but also served as a protecting group for the phenolic hydroxyl group, preventing the formation of additional hydrogen bonds competing with those involving the carboxylic acid centers. At the same time, the long alkyl chains provide additional sites for intermolecular van der Waals interactions that can operate within the assemblies through an interdigitation mechanism.[qv: 3a]

### 2.1 Self‐Assembly of **1** in CHCl_3_

The first solvent tested was purified and dried chloroform (details of the purification procedure can be found in the Supporting Information), in which amino acid–derived NDIs tend to self‐assemble via formation of COOH∙∙∙HOOC hydrogen bonds,^20^ with the resulting polymer structure depending on both steric hindrance and possible *syn*/*anti* conformations of the substituents on the NDI molecule.^20^ Namely, while *S*‐trityl cysteine derivatives tend to self‐assemble into relative short helical nanotubes (with a reported degree of polymerization in 2–15 mer range, at 0.9 × 10^−3^ m and 298 K),[qv: 20b] as a result of the *syn* conformation, the isoleucine[qv: 20c] and C_4_ acylated tyrosine^12^ derivatives adopt *anti* geometry, yielding nanofibrillar structures.

In contrast to the previously published work,^12^ acylation of the tyrosine side chain with long palmitoyl, instead of butyryl substituents, in **1** led to the *syn* conformation and consequently to the loss of intramolecular stacking interactions between the tyrosine and NDI aromatic rings, as established from the ^1^H−^1^H rotating frame overhauser enhancement spectroscopy (ROESY) spectrum (Figure S3, Tables S2 and S3, Supporting Information).

**Figure 2:**
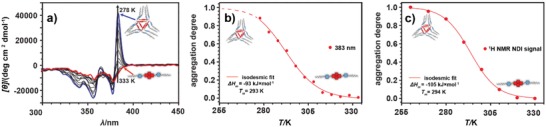
Self‐assembly of **1** into **hel–1*_n_*** in CHCl_3_/CDCl_3_ as followed by CD and ^1^H NMR spectroscopy. a) Variable temperature CD spectra recorded during cooling of a solution of **1** (1.0 × 10^−4^ m, *T* = 333–278 K, cooling rate: −1 K min^−1^). Spectra for the highest and lowest temperatures are marked in red and blue, respectively. b) Normalized temperature‐dependent CD intensity recorded during cooling of a solution of **1** (1.0 × 10^−4^ m, *T* = 333–278 K, cooling rate: −1 K min^−1^). c) Normalized temperature‐dependent ^1^H chemical shift recorded during cooling of a solution of **1** (1.0 × 10^−4^ m, *T* = 330–260 K, cooling rate: −1 K min^−1^). Spectra were referenced on the tetramethylsilane (TMS) resonance at δ = 0.00 ppm (See Figures S7 and S9 in the Supporting Information).

The adoption of the *syn* conformation by **1** in CHCl_3_ suggested the formation of hydrogen‐bonded helical nanotubes (**hel–1*_n_***), which could indeed be inferred from temperature‐dependent circular dichroism (CD) spectroscopy (**Figure** **2**a). The self‐assembly of a helical nanotube was manifested by the appearance of a strong positive Cotton band with a maximum at λ_max_ = 383 nm, in full agreement with earlier CD studies on helical nanotubes;[qv: 20a] the intensity of which was strongly dependent on both temperature and concentration as a result of changes in the degree of aggregation.^21^ Variable temperature (VT) CD spectra of **1** in CHCl_3_ (Figure 2a) revealed that the positive Cotton band at λ_max_ = 383 nm gradually increased in intensity upon cooling. In line with earlier work,[qv: 20b] VT UV–vis spectroscopy revealed that π–π stacking was not occurring during the assembly of **1** into helical nanotubes upon cooling, i.e., no characteristic bathochromic shift, indicative of π–π stacking could be discerned (Figure S16, Supporting Information).

Plotting the maximum CD intensity as a function of temperature and subsequent fitting of the VT CD data revealed an isodesmic supramolecular polymerization model^22^ (Figure 2b), characteristic for helical nanotubes,[qv: 20b] yielding Δ*H*_m_ = −93.4 ± 9.6 kJ mol^−1^ for the enthalpy release during the nanotube formation. The polymerization mechanism was then verified using VT ^1^H NMR, which due to the wider range of available temperatures allowed us to follow the full self‐assembly process from monomer to fully aggregated state. VT ^1^H NMR spectra of **1** in CDCl_3_ (Figure S7, Supporting Information) revealed that the single NDI resonance around δ ≈ 8.6 ppm become more shielded and broadened upon decreasing the temperature (Figure S9a, Supporting Information). Plotting this change as a function of temperature and subsequent fitting confirmed an isodesmic polymerization model for **1** in CDCl_3_, yielding Δ*H*_m_ = −105.8 ± 4.8 kJ mol^−1^ (Figure 2c). This negative enthalpy values indicate that the polymerization is enthalpy driven, which is in agreement with the intermolecular hydrogen bonding proposed to be the main driving force in chloroform. VT ^1^H NMR spectra also proved the presence of additional weak CH∙∙∙O hydrogen bonding interactions between NDI cores, previously observed for the hydrogen‐bonded helical nanotubes.[qv: 20b] Below 260 K, the single NDI resonance split into two peaks (δ = 8.61 and 8.55 ppm; Figure S7, Supporting Information) as a result of the desymmetrization of the NDI core upon formation of side CH∙∙∙O hydrogen bonds.[qv: 20b] Although this effect was quenched at higher temperatures by the fast (on the NMR time scale) exchange between **hel–1*_n_*** and nonaggregated **1**, at low temperatures (and full aggregation) these dynamics were slow enough to observe those weak interactions in the ^1^H NMR measurements.

In addition, at a concentration of 1.0 × 10^−4^ m, a melting temperature, *T*_m_, defined as the temperature at which 50% of the monomers are aggregated, of 293–294 K (from the VT CD and VT NMR) was found for **hel–1*_n_***. Interestingly, the low melting temperature of the **hel–1*_n_*** in CHCl_3,_ compared to the one formed from the *S*‐trityl cysteine–derived NDI (*T*_m_ = 320 K, at 0.9 × 10^−3^ m),[qv: 20b] underlines the effect of the long C_16_ alkyl chains present in **1**, which improve the solubility and thereby reduce the overall stability of the assembly.

The isodesmic self‐assembly mechanism that we observed for **hel–1*_n_***, which is characteristic for helical NDI nanotubes,[qv: 20b] results in rather short assemblies (degree of polymerization typically in the order of 10^0^–10^2^). In line with this, DOSY NMR (diffusion‐ordered spectroscopy) in CDCl_3_ revealed a relatively small radius of 1.2 nm at 298 K (see **Table** **1** and Figure S5, Supporting Information).

**Table 1:** Solvodynamic radii and spherical volumes of assemblies of **1** in CDCl_3_ and MCH‐*d*_14_, as calculated using the Stokes–Einstein equation for the DOSY NMR data recorded at 298 K, and 1.0 × 10^−3^ m (see the Supporting Information for details)

Solvent	*D* [m^2^ s^−1^]	*R*_sol_ [Å]	*V*_sol_ [nm^3^]
CDCl_3_	3.33 × 10^−10^	12.2	7.6
MCH‐*d*_14_	6.72 × 10^−11^	47.8	458

Further evidence for this hydrogen bond‐driven assembly of **hel–1*_n_*** was provided by the observation that addition of tetrahydrofuran (THF) resulted in disassembly of the helical aggregates due to disruption of the hydrogen bonds (Figure S10, Supporting Information). Since full disassembly of **hel–1*_n_*** was observed after addition of about 0.2% of THF, special purification and drying of chloroform was necessary (details of the purification procedure are provided in the Supporting Information), as this solvent has a high tendency to oxidize when exposed to light, and therefore it is often stabilized with highly polar additives (i.e., ethanol).

In conclusion, in full agreement with previous literature reports,^20^, ^21^ and based on a variety of experimental evidence (i.e., ^1^H–^1^H ROESY NMR, (temperature‐dependent) UV–vis and CD spectroscopy, ^1^H NMR spectroscopy, and hydrogen bond competition experiments), it can be summarized that the self‐assembly of **1** in CHCl_3_ yields helical nanotubes (**hel–1*_n_***), similar to those previously reported for *S*‐trityl cysteine NDI derivatives,^20^ as a result of the formation of two types of hydrogen bonds: a) between COOH units and b) weak CH∙∙∙O interactions between NDI rings. The presence of both of these interactions could also be concluded from infrared (IR) spectroscopy on **1**, recorded in CHCl_3_ solution, which revealed bathochromic shifts of the ν(C=O) bands from the COOH and imido groups by 30 and 10 cm^−1^ respectively, in comparison with the spectrum recorded in THF (Figure S23, Supporting Information), along with the ν(O—H) bands from the terminal COOH (unbonded) groups in the nanotube above 3500 cm^−1^ (Figure S25, Supporting Information). In contrast to the experimentally observed hydrogen bonds, in such helical nanotubes, *π–π* interactions are absent, as we could experimentally confirm by ROESY and VT UV–vis spectroscopy.

### 2.2 Self‐Assembly of **1** in Methylcyclohexane

While **1** tends to self‐assemble into **hel–1*_n_*** in CHCl_3_, we anticipated that a decrease of solvent polarity, beyond the range optimal for hydrogen‐bonded helical nanotubes,[qv: 20b] would increase the significance of other noncovalent interactions, which may have a substantial impact on both the assembly mechanism and the final structure of the supramolecular assemblies formed. In fact, thanks to the introduction of long aliphatic C_16_ tails to the structure of **1**, it became possible to study the self‐assembly process in highly nonpolar hydrocarbon‐type solvents (vide infra), which is a unique property in this class of compounds.

To investigate our hypothesis about different self‐assembly outcomes of **1** in nonpolar media, we selected methylcyclohexane (MCH) as solvent, due to both its apolar nature (ε = 2.02, *µ* ≈ 0 D) and suitable melting and boiling points (*T*_m_ = 146 K, and *T*_b_ = 374 K, at *p* = 101.3 kPa) that provides a wide window for variable temperature measurements.

The first evidence for the formation of a different type of supramolecular assembly, compared to that found in CHCl_3_, upon solvent switching was provided by ^1^H NMR spectroscopy. Despite the fact that ^1^H NMR spectra recorded both in CDCl_3_ and MCH‐*d*_14_ maintained the same symmetry, the spectrum recorded in MCH‐*d*_14_ had significantly broadened (full width at half maximum (FWHM) > 300 Hz at Φ_B_ = 16.5 T), suggesting that in MCH much larger assemblies had formed (Figure S4, Supporting Information). This conclusion is in agreement with the results of DOSY NMR, where in contrast to a solvodynamic radius of *R*_sol_ = 1.2 nm for **1** in CDCl_3_ solution, the solvodynamic radius for **1** in MCH‐*d*_14_ solution was calculated to be *R*_sol_ = 4.8 nm (according to the Stokes–Einstein equation, see Table 1 and also Figure S6 in the Supporting Information), which is in line with the further results obtained from variable temperature dynamic light scattering (VT DLS) studies that will be discussed later (vide infra). While a diameter of nearly 10 nm is direct evidence for the formation of a supramolecular polymer of appreciable length (in particular, when considering a typical π‐stacking distance of 0.34 nm), we did not attempt to convert this diameter to an actual stack length, because the spherical shape assumed in DOSY NMR does not accurately describe the 1D supramolecular polymers.

**Figure 3:**
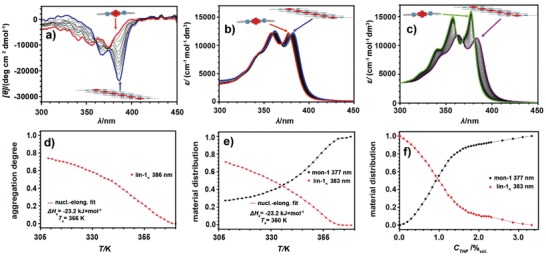
Self‐assembly and disassembly of **lin–1*_n_*** in MCH as followed by UV–vis and CD spectroscopy. a) Variable temperature CD spectra recorded during cooling of a solution of **1** (5.0 × 10^−5^ m, *T* = 370–308 K, cooling rate: −1 K min^−1^). Spectra for the highest and lowest temperatures are marked in red and blue, respectively. b) Variable temperature UV–vis spectra recorded during cooling of a solution of **1** (5.0 × 10^−5^ m, *T* = 370–308 K, cooling rate: −1 K min^−1^). Spectra for the highest and lowest temperatures are marked in red and blue, respectively. c) UV–vis spectra recorded during titration of solution of **1** in MCH with THF (1.0 × 10^−4^ m, *C*_THF_ = 0.0–3.5% v/v). Starting and ending spectra are marked in magenta and green, respectively. d) Normalized temperature‐dependent CD intensity recorded during cooling of a solution of **1** (5.0 × 10^−5^ m, *T* = 370–308 K, cooling rate: −1 K min^−1^). e) Normalized temperature‐dependent UV absorption recorded during cooling of a solution of **1** (5.0 × 10^−5^ m, *T* = 370–308 K, cooling rate: −1 K min^−1^). f) Distribution of monomers over the aggregated (probed at 383 nm) and molecular dissolved state (probed at 377 nm) during titration of solution of **1** with THF (1.0 × 10^−4^ m, *C*_THF_ = 0.0–3.5% v/v).

Variable temperature CD analysis further confirmed the formation of a morphologically completely different polymer in MCH (**Figure** **3**a). The registered Cotton band in CD was not only bathochromically shifted by ≈3 nm, but also the molar ellipticity had a negative sign, which indicates a dramatic change in the angle between NDI units and the adoption of a planar conformation of **1** in MCH solution (Figure 1).^21^, ^23^

The adoption of a planar conformation enables the formation of *π–π* stacking interactions between the NDI and tyrosine aromatic rings. Although the overall width of the ^1^H NMR signals of **1** in MCH‐*d*_14_ prevented the registration of overhauser effects, the formation of the *π–π* stacking interactions was observed in the UV–vis spectra, as indicated by the bathochromic shift (≈6 nm) of the main absorption band during cooling of the solution of **1** in MCH (Figure 3b), as well as by the reverse hypsochromic shift (≈6 nm) observed during the solvent‐driven disassembly of **1** in MCH, induced by the addition of highly polar THF to the solution (Figure 3c). It is noteworthy to point out that upon addition of THF, the transformation from supramolecular aggregate to monomer occurred in a nonlinear fashion (Figure 3f), and was already completed after addition of ≈2.5% of THF.

In contrast to the noncooperative, isodesmic assembly of **hel–1*_n_*** in CHCl_3_, the VT UV–vis and VT CD data for **1** in MCH revealed a clear nonsigmoidal transition (Figure 3d,e), indicative for a cooperative supramolecular polymerization mechanism, which can be characterized by an initial unfavorable nucleation step, followed by a more favorable elongation step.[qv: 1a] Both the VT UV–vis and VT CD cooling plots could be fitted using a nucleation–elongation model for cooperative supramolecular polymerization,^24^ yielding Δ*H*_e_ = −23.2 ± 0.7 kJ mol^−1^ with *T*_e_ = 360 K and dimensionless activation constant *K*_a_ = 1.3 × 10^−5^ based on the VT UV–vis data, and Δ*H*_e_ = −23.2 ± 0.7 kJ mol^−1^ with *T*_e_ = 366 K based on the VT CD data, respectively (Figures S11 and S12, Supporting Information). The lack of a clear nucleation regime in the VT CD plot made it impossible to determine the *K*_a_ from this experiment. To better study the nucleation step, and to confirm the nonisodesmic, cooperative assembly mechanism, VT CD data were also recorded at lower concentration (2.5 × 10^−5^ m; see Figure S13 in the Supporting Information), as to shift the transition to a lower temperature range, accessible for the VT unit. Indeed as expected, lowering the concentration shifted the *T*_e_ down to 342 K, yielding Δ*H*_e_ = −23.6 ± 1.6 kJ mol^−1^, as well as a clear nucleation step with *K*_a_ = 5.2 × 10^−5^.

While the elongation enthalpy Δ*H*_e_ as determined from the VT UV–vis and VT CD studies at 5.0 × 10^−5^ m is equal, the 6 K difference in the elongation temperatures *T*_e_, which is the temperature at which the polymerization (elongation) starts, was observed. Although this difference could be partially attributed to experimental error resulting from measuring the UV–vis and CD data on separate spectrometers, it most likely points to a genuine difference in the onset temperature at which the assembly starts to happen. In addition, due the cooperative nature of the self‐assembly process, the assembly might not be under full equilibrium at a cooling rate of −1 K min^−1^, which could further contribute to the observed difference in elongation temperature *T*_e_.

The data recorded in MCH indicate that the reduction of the solvent polarity not only changed the global morphology of the assembly, leading to the formation of a linear, *π–π* stacking polymer (**lin–1*_n_***), at the expense of the nanotube, but it also moved the supramolecular polymerization from a simple isodesmic into a more complicated cooperative model, opening the way to more complex assembly behavior.

**Figure 4:**
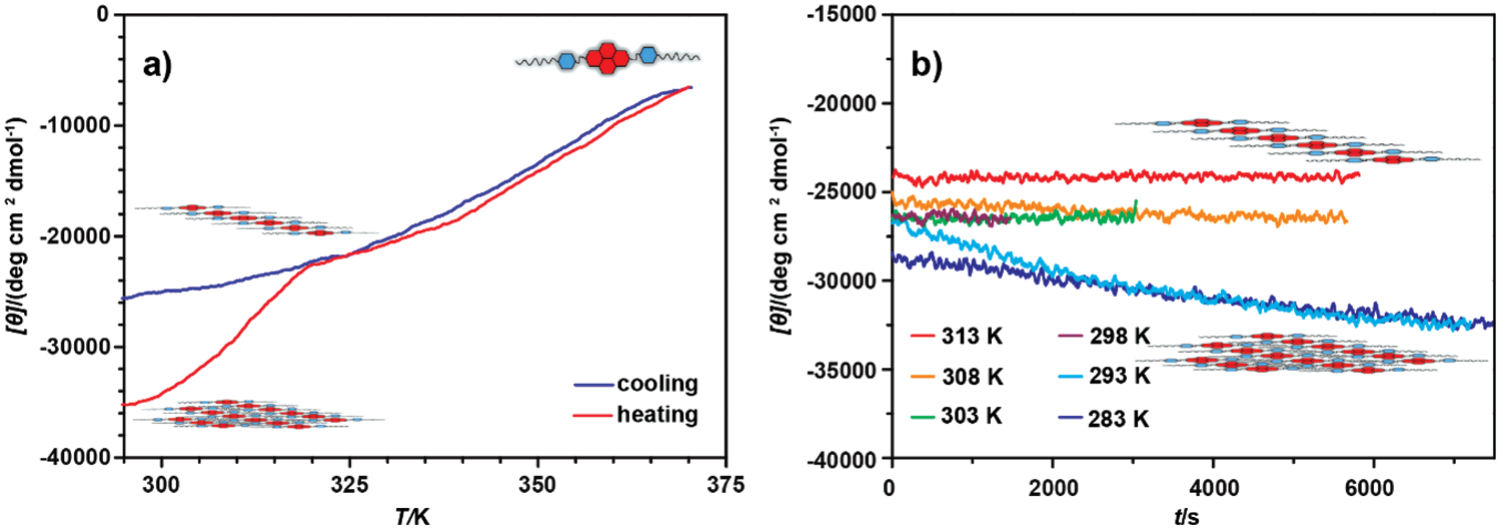
Bundling of the **lin–1*_n_*** into **agg–1*_n_*** in MCH followed by variable temperature CD. a) Disassembly and self‐assembly of **1** in MCH (5.0 × 10^−5^ m) followed by variable temperature CD at λ = 386 nm (*T* = 370–295 K, heating/cooling rate: ±1 K min^−1^). First the freshly prepared sample was heated and then cooled again. b) Time‐dependent evolution of the CD intensity (λ = 386 nm) after cooling a sample of **1** in MCH (5.0 × 10^−5^ m) to the specified temperature and subsequently leaving it at that temperature while monitoring the CD intensity.

In an attempt to further understand the cooperative self‐assembly of **lin–1*_n_*** in MCH, we recorded both the heating and cooling curves by CD spectroscopy (**Figure** **4**a). From the VT CD cooling and heating plots, two distinct stages of self‐assembly could be identified. That is, above 320 K, the assembly of **lin–1*_n_*** was under thermodynamic control (for a heating/cooling rate of ±1 K min^−1^). In contrast, below 320 K a large difference in the heating and cooling curves was found, suggesting that when the temperature drops below 320 K, a cooling rate of −1 K min^−1^ is no longer sufficiently slow to keep the continued self‐assembly of **lin–1*_n_*** under thermodynamic control, resulting in a clear hysteresis between the heating and cooling curves. This implies that a second type of aggregate, with drastically slower equilibration kinetics had formed in this second stage, once a certain threshold temperature had been passed.

We could rule out that this second stage is the result of precipitation of the monomer, based on the following observations: 1) during the entire cooling run, an isosbestic point could be seen at 370 nm in the UV–vis spectrum of the solution (Figure S17, Supporting Information); 2) no Tyndall/Rayleigh scattering effects could be discerned in the (red part of the) UV–vis spectra, which would be indicative for the presence of insoluble particles in solution (Figure S17, Supporting Information); 3) the cooling/heating runs were fully repeatable; 4) UV–vis spectra recorded at different concentrations followed the Lambert–Beer law; 5) the UV–vis/CD spectra have been recorded at concentrations that are >500 times lower than the determined solubility limit of **1** in MCH (≈3.0 × 10^−2^ m).

While the onset of hysteresis (at ≈320 K) between the heating and cooling curves in Figure 4 seems rather abrupt (which could be indicative of a cooperative transition), we cannot draw this conclusion with certainty; the VT CD data that show the hysteresis are (evidently) the result of nonequilibrium assembly, which implies that the shape of the (temperature‐dependent) CD curve cannot be used directly to deduce the characteristics of the assembly process (because, e.g., the results could be cooling/heating rate dependent).

When we define the threshold temperature as the temperature above which a cooling rate of −1 K min^−1^ results in a thermodynamically controlled self‐assembly, and below which this cooling rate is no longer sufficiently slow to maintain equilibrium, then it becomes possible to pinpoint this threshold temperature, *T*_th_. That is, below this temperature, a sample cooled with −1 K min^−1^ is out of equilibrium, meaning that when the cooling is stopped at a specific temperature (lower than *T*_th_), the assembly process will continue as the sample still has to reach equilibrium. This convergence toward equilibrium should be seen in CD data by the continued change in CD intensity once the cooling is stopped (see Figure S17 in the Supporting Information for the corresponding UV spectra).

To this end, a solution of **1** in MCH was cooled down from 370 K to a specified temperature in the range of 318–283 K, after which it was left at the specific temperature for 1–2 h, while the CD signal was monitored at the same time. Between each run, the sample was reheated to 370 K to avoid any “memory effect.” As can be seen in Figure 4b, when the sample was cooled down from the molecularly dissolved state to a temperature above 298 K, the CD intensity remained constant when the sample had reached the specified temperature and was subsequently left at this temperature. In that case, when reheating this sample to 370 K, no hysteresis in the cooling and heating curves was observed. In contrast, when initially cooling (with −1 K min^−1^) to a temperature equal to, or lower than, 298 K, the polymer thus formed was no longer at equilibrium as evidenced form the decrease in CD intensity (note that the CD intensity is negative in sign) when leaving the sample at the specified temperature. Reheating after 1 h at this low temperature also yielded a heating curve that was different from the cooling curve (Figure S14, Supporting Information).

Based on these observations, we propose that upon cooling of the solution of **1** from 370 K, at which **1** is in the monomeric (**mon–1**) state, initially a 1D supramolecular polymer **lin–1*_n_*** is formed under thermodynamic control when the cooling rate is −1 K min^−1^. However, when the temperature is reduced to below 298 K, these **lin–1*_n_*** assemblies form a second, higher‐order aggregate, e.g., bundling of individual polymers takes place. These bundles (**agg–1*_n_***) are no longer under equilibrium, i.e., when halting the cooling, the aggregation/bundling continues.

**Figure 5:**
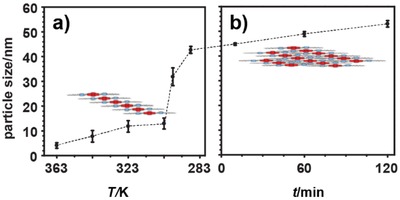
Bundling of the **lin–1*_n_*** into **agg–1*_n_*** in MCH followed by variable temperature dynamic light scattering (VT DLS) analysis. a) Average particle size as a function of temperature, as obtained during cooling of a solution of **1** in MCH (1.0 × 10^−3^ m, *T* = 363–288 K, equilibration time *t*_eq_ = 30 min; see also Figure S15 in the Supporting Information for full spectra). b) DLS kinetic plot recorded at 288 K after cooling of a solution of **1** from 368 K (1.0 × 10^−3^ m). The average value and standard deviation from six experiments are shown.

Evidence for the proposed transition from linear 1D polymers (**lin–1*_n_***) to bundled higher‐order aggregates (**agg–1*_n_***) was obtained by VT DLS (see **Figure** **5**). To this end, a solution of **1** in MCH was heated up to 363 K, and after 2 h the solution was gradually cooled down to 288 K, while halting the cooling at a number of intermediate temperatures to assess the size of the self‐assembled polymer by DLS. As shown in Figure 5a, in the initial cooling stages, at (moderately) high temperatures, the formation of small objects with a solvodynamic diameter of ≈10 nm was observed, which can be attributed to the formation of a **lin–1*_n_*** polymer, in accordance with the previously presented DOSY NMR results where a solvodynamic diameter of 9.6 nm was observed (*R*_sol_ = 4.8 nm; see Table 1). However, when this solution was cooled down below 303 K, a sharp increase in particle size was observed, reaching a diameter of about 45 nm at 288 K, which supports the proposed transition of **lin–1*_n_*** into a aggregated/bundled assembly, **agg–1*_n_***.

After this sharp increase in particle size, the sample was left at 288 K for 2 h to monitor any further growth of the assemblies (Figure 5b). Indeed, in agreement to the kinetic transition observed at low temperature by CD (Figure 4b), also by DLS, further aggregation was observed which ultimately yielded **agg–1*_n_*** assemblies with a diameter of ≈55 nm.

Although the higher concentration used in DLS analysis was found to shift the *T*_th_ up to 303 K and accelerated the kinetics of the bundling process, the VT DLS results are fully in line with the VT CD data and, at the same time, confirmed that the self‐assembly of **1** in MCH is indeed a two‐stage process involving formation of a linear **lin–1*_n_*** polymer followed by its aggregation into a multidimensional **agg–1*_n_*** superstructure.

IR spectra of **1** recorded in MCH solution (at 3.0 × 10^−2^ m; see Figure S23 in the Supporting Information) revealed that in the **agg–1*_n_*** the COOH groups become involved in the formation of hydrogen bonds, as manifested by the bathochromic shift of the ν(C=O) band by 30 cm^−1^. Based on this observation, we propose that hydrogen bonding leads to the bundling of the individual stacks of **lin–1*_n_***, as observed in DLS. In addition, as a result of the interstack hydrogen bonds, the entire aggregate, **agg–1*_n_***, might become more rigid, which could lead to the slower assembly kinetics, as observed with VT CD spectroscopy. In contrast to the helical nanotubes in CHCl_3_, the influence of the side CH∙∙∙O hydrogen bonds was found to be negligible for **agg–1*_n_*** (Figure S23, Supporting Information). Unfortunately, attempts to further characterize these aggregates by atomic force microscopy (AFM), or other surface‐based methods, failed due to the disruption of the assemblies upon surface immobilization (see Figure S22 in the Supporting Information for representative AFM results).

**Figure 6:**
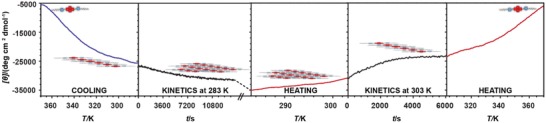
Multistep thermodynamics and kinetics of disassembly and self‐assembly of **1** in MCH (5.0 × 10^−5^ m) followed by circular dichroism at λ = 386 nm. Cooling, heating, and kinetic plots are marked in blue, red, and black, respectively. All heating and cooling steps were performed with a rate of ±1 K min^−1^. During the kinetic measurement, the sample was left at the indicated temperature for the specified amount of time. The cartoon in each plot indicates the major species present under those conditions.

To get deeper insight into this two‐stage assembly process involving thermodynamic and kinetic products, we designed a five‐step experiment in which we set out to observe all thermodynamic and kinetic stages of the assembly and disassembly of **1** (**Figure** **6**). After heating the solution of **1** to 370 K (and leaving it to fully equilibrate at this temperature for 60 min), the sample was slowly cooled down to 283 K (at a rate of −1 K min^−1^) to once again follow the self‐assembly process. As before, the polymerization of **1** into **lin–1*_n_*** in MCH followed the cooperative model, but the aggregation did not stop when the temperature reached 283 K (as this temperature is below the previously determined threshold temperature) and the **lin–1*_n_*** polymer continued to assemble into larger **agg–1*_n_*** structures, as evidenced by the further time‐dependent decrease in CD intensity (see the second panel of Figure 6). After 24 h, at 283 K the sample was fully stabilized, after which the **agg–1*_n_*** polymer was slowly reheated up to 303 K. From previously recorded cooling data (Figure 4b, green plot) we could infer that at this temperature only the nonbundled, **lin–1*_n_*** assemblies are stable. Indeed, leaving the sample at this temperature, induced the slow, time‐dependent debundling of the **agg–1*_n_***, as evidenced in a reduction of the magnitude of the CD effect (see the fourth panel of Figure 6). After about 1 h the kinetic debundling process ended, which should leave the **lin–1*_n_*** polymer in solution. Further heating of this solution to the molecularly dissolved (**mon–1**) species revealed a mirror image of the cooling plot (Figure 6, first panel), confirming that indeed only the **lin–1*_n_*** equilibrium polymer was still in solution.

### 2.3 The Competition between Polymers

**Figure 7:**
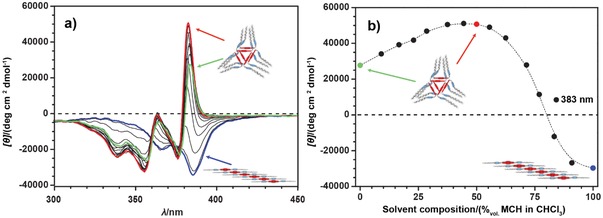
a) CD spectra recorded during titration of a solution of **1** in CHCl_3_ with a solution of **1** in MCH (both solutions at 1.0 × 10^−4^ m and 298 K). The spectra evolved from green (0% MCH) via red (50% MCH) to blue (100% MCH). b) Changes in the CD intensity upon titration of **1** solution in CHCl_3_ with the solution of **1** in MCH (both solutions at 1.0 × 10^−4^ m and 298 K). The colored markers in the graph correspond to the full CD spectra of the same color. Note: in this experiment, the kinetic formation of **agg–1*_n_*** has been intentionally prevented by a combination of temperature and the time of the measurement.

After detailed investigation of the thermodynamics and kinetics of the formation of separate assemblies from **1** in CHCl_3_ and MCH, we decided to establish whether by a proper control over the polarity of the system, one might be able to direct the polymerization of **1** toward the desired assembly type using solvent as an external stimulus.[qv: 15b] In the first instance, we decided to determine the concentration of CHCl_3_ in MCH at which the transition between the linear polymer (**lin–1*_n_***, stable in pure MCH) and helical assembly (**hel–1*_n_***, stable in pure CHCl_3_) can be observed. As shown in **Figure** **7**, the helical assembly, **hel–1*_n_***, is stable up to ≈50% of MCH, as indicated by the positive CD effect up to this concentration. In fact, in this range, the reduction of the solvent polarity seems to have a positive effect on the **hel–1*_n_*** stability as can be inferred from the increase in CD intensity. Although we note that this increase could also be the result of an increase in the assembly's intrinsic molar ellipticity value upon the reduction in polarity. In contrast, a further decrease beyond 50% in solvent polarity weakens the **hel–1*_n_*** structure, leading to a smooth transition into the **lin–1*_n_*** polymer, as indicated by the negative CD effect above 75% of MCH. Those fluctuations in the stability of the helical nanotube upon changes in solvent composition were observed previously for related *S*‐trityl cysteine–derived NDI nanotubes.[qv: 20b] However, in contrast to the previous studies, the design of **1** allows the molecules to reassemble into different linear assemblies that can be formed in less polar media.

**Figure 8:**
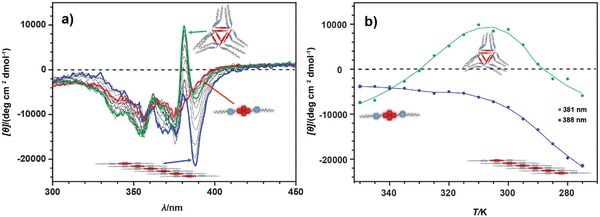
a) VT CD spectra of **1** in MCH:CHCl_3_ mixture (75:25, v/v) (1.0 × 10^−4^ m, *T* = 353–278 K, cooling rate: −1 K min^−1^). Spectra recorded at 353, 313, and 278 K marked in red, green, and blue, respectively, which correspond to the **mon‐1**, **hel–1*_n_***, and **lin–1*_n_***, respectively, as major species (see also Figure 7A). b) Temperature‐dependent CD intensity recorded at a wavelength characteristic for formation of **hel–1*_n_*** (381 nm) and **lin–1*_n_*** (388 nm), during cooling of **1** solution (1.0 × 10^−4^ m, *T* = 353–278 K, cooling rate: −1 K min^−1^).

Knowing the difference in the thermal stability of both thermodynamic polymers (i.e., **hel–1*_n_*** and **lin–1*_n_***), we prepared a mixture halfway between the most stable **hel–1*_n_*** (at 50/50 MCH/CHCl_3_) and **lin–1*_n_*** (at 100% MCH). VT CD cooling spectra of **1** recorded in this mixture, consisting of 75% of MCH and 25% of CHCl_3_ (**Figure** **8**a), revealed that the interplay between the temperature and solvent polarity initially leads to the formation of the helical nanotube assembly (**hel–1*_n_***) from the monomer state (**mon–1**) with the maximum of the positive CD effect at 313 K (Figure 8a, green spectrum). Namely, down to this temperature, the nonlinear, sigmoidal growth of the positive Cotton band indicative for **hel–1*_n_*** was observed (Figure 8b, green trace) with almost no changes in the negative Cotton band originating from **lin–1*_n_*** (Figure 8b, blue trace). However, upon further cooling of the sample, the CD band corresponding to **hel–1*_n_*** gradually disappeared, with concomitant appearance of the CD band for the linear *π–π* stacking polymers (**lin–1*_n_***, Figure 8b), ultimately yielding the stable **lin–1*_n_*** polymer at 278 K (Figure 8a, blue spectrum).

Speculating on the overall mechanism of self‐assembly, we note that the increased polarity of the medium in combination with the more complicated cooperative polymerization mechanism of **lin–1*_n_*** compared to the “simple,” non‐nucleated, isodesmic for **hel–1*_n_*** initially seems to favor the formation of the smaller, isodesmic assembly. However, when the temperature is low enough to overcome the unfavorable nucleation process, **1** reassembles completely into the thermodynamically favored cooperative **lin–1*_n_*** polymer.

By careful selection of the correct solvent mixture, we were able to design a supramolecular system that by simple control over temperature can access three distinct states: a monomeric state (**mon–1**), an isodesmic discrete helical assembly (**hel–1*_n_***), and a cooperative linear polymer (**lin–1*_n_***). Further investigations have shown that although the polarity of this mixture is low enough to reassemble the **hel–1*_n_*** into **lin–1*_n_***, it is still too high to induce the kinetic bundling of **lin–1*_n_*** into **agg–1*_n_***. Namely, leaving this solution even at 263 K (Figure S18, Supporting Information) did not show any further changes of the CD intensity, which would be indicative for the formation of **agg–1*_n_*_._**

## 3 Conclusion

We showed that one relatively simple compound can create several noncovalent supramolecular structures differing not only in the global morphology, but also in the thermodynamics and kinetics of their formation. Namely, while the self‐assembly of **1** in chlorinated solvents led to the formation of the previously known helical nanotubes **hel–1*_n_*** whose self‐assembly is driven by an isodesmic polymerization model; in solvents of lower polarity, larger and structurally more complex polymers were observed. For the latter type of assemblies, the interplay between H‐bonding, *π–π* stacking, and van der Waals interactions yielded noncovalent cooperative polymers **lin–1*_n_*** and **agg–1*_n_*** with noticeable hysteresis on both formation and deformation processes. Ultimately, the formation of three morphologically and thermodynamically distinct noncovalent assemblies generated from a single molecule could be achieved by careful selection of the external conditions. Moreover, the obtained insights into each of the two solvents, chloroform and MCH, also allowed us to select the correct solvent mixture to design a supramolecular system, that by simple control over temperature can access three distinct states: monomer, nanotube, and *π–π* stacking polymers.

In conclusion, our work shows that in the noncovalent assembly even the most subtle changes in the environment can have a tremendous impact on the self‐assembly outcome. That is, a well‐understood, both by structural and physicochemical means, group of compounds such as amino acid derivatives of naphthalene diimides still can undergo rather complex assembly processes by the very delicate nature of the interactions that occur.

## Conflict of Interest

The authors declare no conflict of interest.

## Supporting information

Supplementary
