# Supplementary material for: Steering the Self‐Assembly Outcome of a Single NDI Monomer into Three Morphologically Distinct Supramolecular Assemblies, with Concomitant Change in Supramolecular Polymerization Mechanism
Source: Adv Sci (Weinh). 2019 Jun 14;6(16):1900577. doi: 10.1002/advs.201900577 (PMC6702645; doi:10.1002/advs.201900577)
Supplement: Supplementary file 1 — Supplementary [file ADVS-6-1900577-s001.pdf]

Copyright WILEY-VCH Verlag GmbH & Co. KGaA, 69469 Weinheim, Germany, 2019.

ADVANCED SCIENCE Open Access

Supporting Information

for *Adv. Sci.,* DOI: 10.1002/advs.201900577

Steering the Self-Assembly Outcome of a Single NDI Monomer into Three Morphologically Distinct Supramolecular Assemblies, with Concomitant Change in Supramolecular Polymerization Mechanism

Grzegorz Markiewicz, Maarten M. J. Smulders, and Artur R. Stefankiewicz**

Copyright WILEY-VCH Verlag GmbH & Co. KGaA, 69469 Weinheim, Germany, 2018.

Supporting Information

Steering the Self-Assembly Outcome of a Single NDI Monomer into Three Morphologically Distinct Supramolecular Assemblies, with Concomitant Change in Supramolecular Polymerization Mechanism

Grzegorz Markiewicz, Maarten M. J. Smulders, and Artur R. Stefankiewicz**

E-mail: maarten.smulders@wur.nl, ars@amu.edu.pl

Content

1. General S2
2. Data processing S3
3. Synthesis S5
4. NMR data S7
5. Supplementary spectra and plots S11
6. Atomic Force Microscopy S18
7. FT-IR S19
8. References S21

1. General

Chemicals were purchased from commercial suppliers (mainly Sigma Aldrich and Merck) and used as received. Solvents used for spectroscopic analysis were purchased at ACS spectroscopic grade (Sigma Aldrich). Methylcyclohexane and THF were used as received. Chloroform was purified and dried prior to use as follows: Commercial $CHCl_3$ was extracted 3 times with equal volumes of deionized water, pre-dried with anhydrous potassium carbonate, filtered, and then dried with the activated Al_2O_3 (Basic Brockmann ≈ 15% of the $CHCl_3$ volume) for 24 h in an amberized bottle.

NMR solvents were purchased from Deutero GmbH (Germany). $CDCl_3$ was purchased with silver foil stabilizer, and dried with the activated Al_2O_3 (Basic Brockmann, ≈ 15% of the $CDCl_3$ volume) prior to use. MCH-d_{14} was purchased in single-use ampoules and used as received. NMR spectra were recorded on a Bruker Fourier 300 MHz, Bruker Avance III HD 600 MHz, or Bruker Avance III 700 MHz spectrometers and referenced on TMS $\delta = 0.00$ ppm.

ESI-MS spectra were recorded on Waters Synapt G2-S Q-TOF mass spectrometer in negative ion mode.

The UV-Vis spectra were recorded on Agilent Cary 100 spectrophotometer equipped with water-loop temperature controller, using 10 mm optical path quartz cuvettes.

The CD spectra were recorded on a Jasco J-710 or Jasco J-810 spectropolarimeter equipped with Peltier-type temperature controller, using 10 mm optical path quartz cuvettes.

The DLS spectra were recorded on Malverin Pananalytical Zetasizer equipped with Peltier-type temperature controller, at 173° scattering angle using 10 mm optical path quartz cuvettes. Samples were filtered 3 times with 0.45 μm PTFE syringe filters prewashed with MCH prior to DLS analysis.

AFM images were recorded on Asylum MFP-3D microscope in AC mode. SiO_2 surfaces were hydrophobized by Me_3SiCl vapors. Samples were deposited on the surfaces by drop-casting method, followed by the spontaneous evaporation of the solvent at room temperature.

FT-IR spectra were recorded on Bruker IFS 66v/S vacuum FT-IR spectrometer in the airtight Si-crystal cuvette, and then processed in Bruker OPUS software. Spectra of the pure solvents were used for subtraction.

2. Data processing

UV spectroscopy

All spectra were replotted and rescaled from Cary WinUV files, using OriginPro 8.0. No data smoothening or noise reduction protocols were applied. After normalization, VT-UV data were fitted to the isodesmic[S1] and nucleation-elongation[S2] models.

CD spectroscopy

All spectra were replotted and rescaled from Jasco Spectra Manager files using, OriginPro 8.0. After normalization, VT CD data were fitted to the isodesmic[S1] and nucleation-elongation[S2] models. For clarity of the figures, spectra and plots given in main text have been smoothen using Savitzky-Golay algorithm, however the thermodynamic parameters were calculated based on the raw data, prior to smoothening.

DLS

Dynamic viscosity of MCH at specified temperatures were obtained from the from Celsius company datasheet (http://www.celsius-process.com), and are listed in Table S1.

Table S1. Dynamic viscosity of MCH as a function of temperature.

Temperature [K]	Dynamic viscosity [mPa × s^{-1}]
363	0.352
343	0.411
323	0.500
303	0.634
298	0.678
288	0.783

All spectra were replotted from Malverin DLS software files using OriginPro 8.0. Each acquisition was repeated 6 times and the average value was provided along with the SD_{mean}. No data smoothening or further processing was applied. The object sizes were calculated using Stokes-Einstein model, assuming that the assemblies are spherical.

DOSY NMR

Spectra were recorded using Bruker *LED* pulse sequence and then replotted from TopSpin files with Mnova 11.0 software using Bayesian DOSY transformation. The solvodynamic radii were calculated using standard Stokes-Einstein equation $D = \frac{k_B\,T}{6\pi\eta R}$, assuming that the nanoparticles are spherical. Due to the lack of viscosity data for MCH-d_{14}, viscosities of nondeuterated solvents at 298 K were used for this calculation, as follows:

- MCH $\eta_{298\,K} = 0.678$ mPa × s^{-1}
- $CHCl_3$ $\eta_{298\,K} = 0.537$ mPa × s^{-1}

NMR spectroscopy

Variable temperature NMR spectra were recorded on a Bruker Avance III HD 600 MHz spectrometer equipped with external BCU II temperature controller. Standard ^{1}H NMR pulse sequence (*zg*) has been used, with Lorentzian line broadening *lb* = 2.0 Hz. Spectra were referenced on TMS δ = 0.00 ppm. After normalization, chemical shifts were fitted to the isodesmic model.[S1]

3. Synthesis

Scheme S1. Overview of the three-step synthesis of **1**.

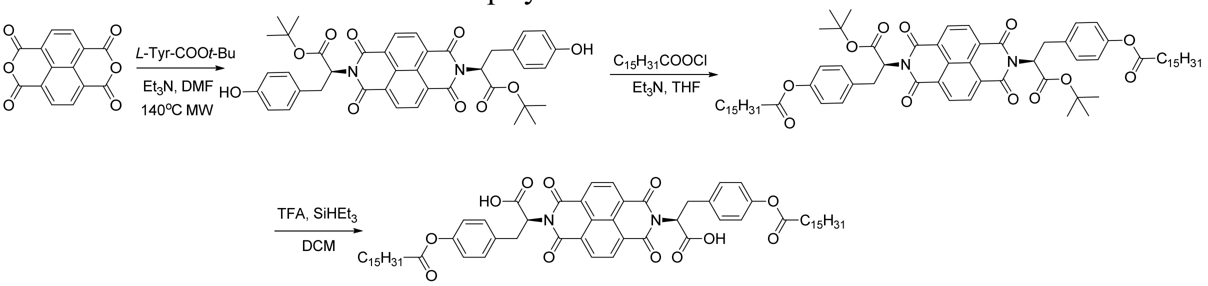

Synthesis of NDI-[*L*-Tyr(OH)COO*t*-Bu]$_2$ 1,4,5,8-naphthalenetetra-carboxylic acid dianhydride (200 mg, 0.745 mmol), *L*-tyrosine *tert*-butyl ester (354 mg, 1.49 mmol) and triethylamine (500 µL) were dissolved in 7 mL of dry DMF. The reaction mixture was heated under microwave irradiation (CEM Microwave reactor) for 5 min at 140 °C. Solvent removal under reduced pressure yielded a dark brown oily residue. The residue was dissolved in 10 mL of MeCN and then added dropwise under stirring to 100 mL of 1M HCl. The resulting precipitate was filtered off, rinsed with 150 mL of deionized water and dried *in vacuo*. The pure product was isolated as an orange powder in 82% yield (430 mg).

^{1}H NMR (300 MHz, CD_3CN) δ (ppm): 8.61 (s, 4H), 6.96 (d, *J* = 8.5 Hz, 4H), 6.68 (s, 2H), 6.51 (d, *J* = 8.6 Hz, 4H), 5.81 (dd, *J* = 10.2, 5.3 Hz, 4H), 3.49 (dd, *J* = 14.3, 5.3 Hz, 2H), 3.26 (dd, *J* = 14.3, 10.2 Hz, 2H), 1.38 (s, 18H).

^{13}C NMR (75 MHz, DMSO-d_6) δ (ppm): 167.76, 161.94, 155.66, 131.25, 129.92, 127.63, 126.06, 125.68, 114.98, 81.35, 55.48, 33.47, 27.54.

Synthesis of NDI-[*L*-Tyr(OCOC$_{15}$H$_{31}$)COO*t*-Bu]$_2$ To the solution of NDI-[*L*-Tyr(OH)COO*t*-Bu]$_2$ (200 mg, 0.28 mmol) in dry THF (30 mL), triethylamine (200 µL, 1.44 mmol) and palmitoyl chloride (220 mg, 0.8 mmol) were added under Ar atmosphere. The mixture was stirred at RT for 12 h. After this time, the solvent was removed under reduced pressure and the resulting paste was dissolved in 30 mL of DCM. The solution was washed with 1M HCl (2 × 25 mL) and deionized water (2 × 25 mL), dried with anhydrous $MgSO_4$ and evaporated. The thus obtained red product was used in the next step without further purification.

^{1}H NMR (300 MHz, $CDCl_3$) δ (ppm): 8.67 (s, 4H), 7.20 (d, J = 8.5 Hz, 4H), 6.86 (d, J = 8.5 Hz, 4H), 5.93 (dd, J = 9.8, 5.7 Hz, 2H), 3.69 (dd, J = 14.4, 5.7 Hz, 2H), 3.46 (dd, J = 14.4, 10.0 Hz, 2H), 2.46 (t, J = 7.5 Hz, 4H), 1.67 (t, J = 7.2 Hz, 4H), 1.30 – 1.20 (m, ≈50H), 0.92 – 0.86 (m, 10H).

^{13}C NMR (75 MHz, $CDCl_3$) δ (ppm): 174.16, 172.21, 167.97, 162.44, 149.49, 134.84, 131.29, 130.12, 126.79, 126.31, 121.56, 82.68, 55.60, 32.03, 29.85-29.65 (overlapped), 29.56, 29.50, 29.37, 29.30, 29.26, 29.17, 28.97, 28.03, 22.79, 14.22.

Synthesis NDI-[*L*-Tyr($OCOC_{15}H_{31}$)COOH]$_2$ (1) To the solution of NDI-[*L*-Tyr($OCOC_{15}H_{31}$)COO*t*-Bu]$_2$ (as obtained in previous step) in DCM (2 mL), trifluoroacetic acid (600 µL, 7.8 mmol) and triethylsilane (120 µl, 0.75 mmol) were added, and reaction mixture was stirred at room temperature for 12 h. Afterwards, the solvents were evaporated under reduced pressure and the resulting paste was dissolved in acetone (5 mL). This solution was added dropwise under vigorous stirring to 50 mL of water. The resulting precipitate was filtered off, rinsed with 25 mL of water and dried over-night in high vacuum. The solid was redissolved in cyclohexane and filtered over a 10 cm celite plug. The filtrate was evaporated and dried *in vacuo*. The pure product was obtained as a brown powder in overall 55% yield (231 mg).

^1H NMR (600 MHz, $CDCl_3$) δ (ppm): 8.57 (s, 4H), 7.17 (d, J = 8.2 Hz, 4H), 6.86 (d, J = 8.1 Hz, 4H), 6.03 (dd, J = 9.8, 5.7 Hz, 2H), 3.71 (dd, J = 15.0, 5.4 Hz, 2H), 3.46 (dd, J = 14.7, 9.9 Hz, 2H), 2.44 (t, J = 7.5 Hz, 4H), 1.69 – 1.64 (m, 4H), 1.26 – 1.23 (m, ≈50H), 0.90 – 0.85 (m, 10H).

^{13}C NMR (150 MHz, $CDCl_3$) δ (ppm): 175.45, 172.47, 162.26, 149.62, 134.13, 131.57, 130.20, 126.77, 126.11, 121.76, 54.51, 34.45, 34.09, 32.07, 29.90-29.70 (overlapped), 29.59, 29.51, 29.37, 29.21, 24.99, 24.76, 22.84, 14.28.

ESI-MS: *m/z* calcd for: $[M–H]^-$, 1070.583, 1069.579, found: 1070.593, 1069.578, calcd for: $[M–2H]^{2–}$ 534.788, 534.286, found: 534.785, 534.290.

UV (THF, $cm^{-1} \times mol^{-1} \times dm^3$) ε_{379} = 19480, ε_{359} = 16910, ε_{342} = 10300, ε_{323} = 5516, ε_{236} = 30740.

4. NMR data

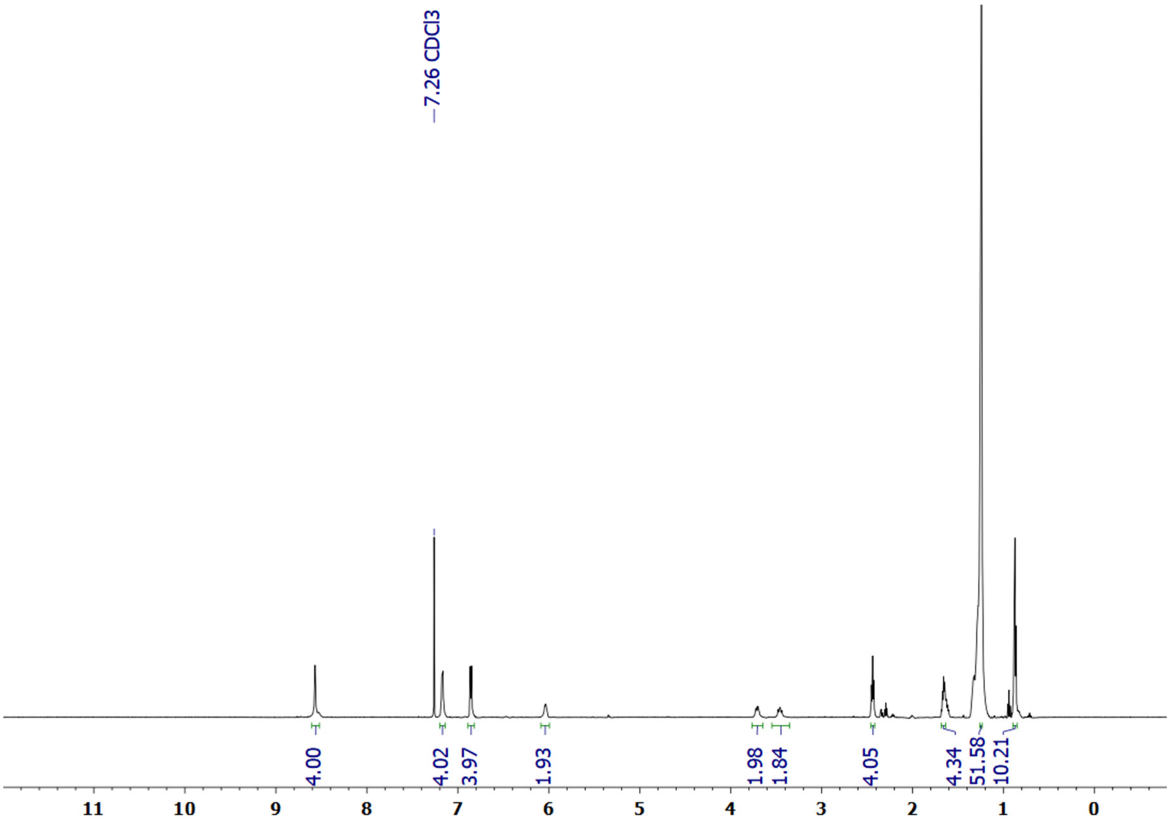

Figure S1. ^{1}H NMR (600 MHz, 298 K, $CDCl_3$) spectrum of **1**.

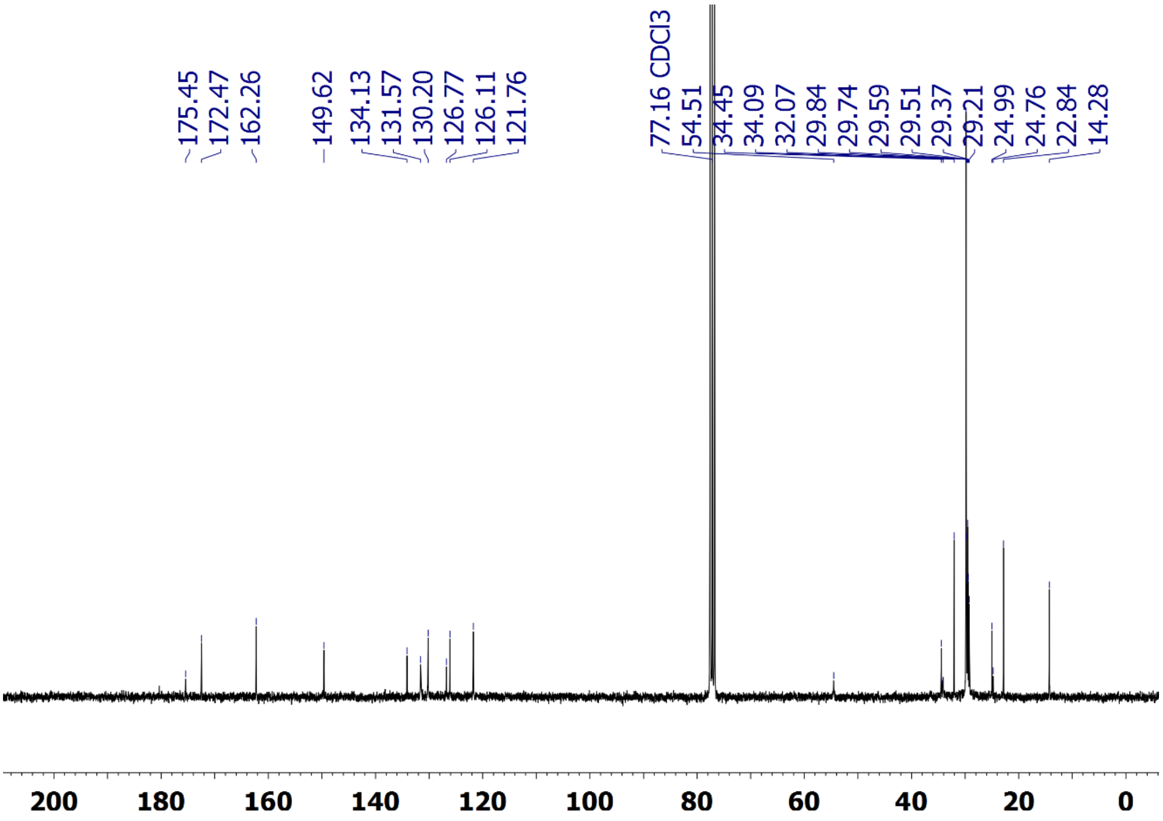

Figure S2. ^{13}C NMR (150 MHz, 298K, $CDCl_3$) spectrum of **1**.

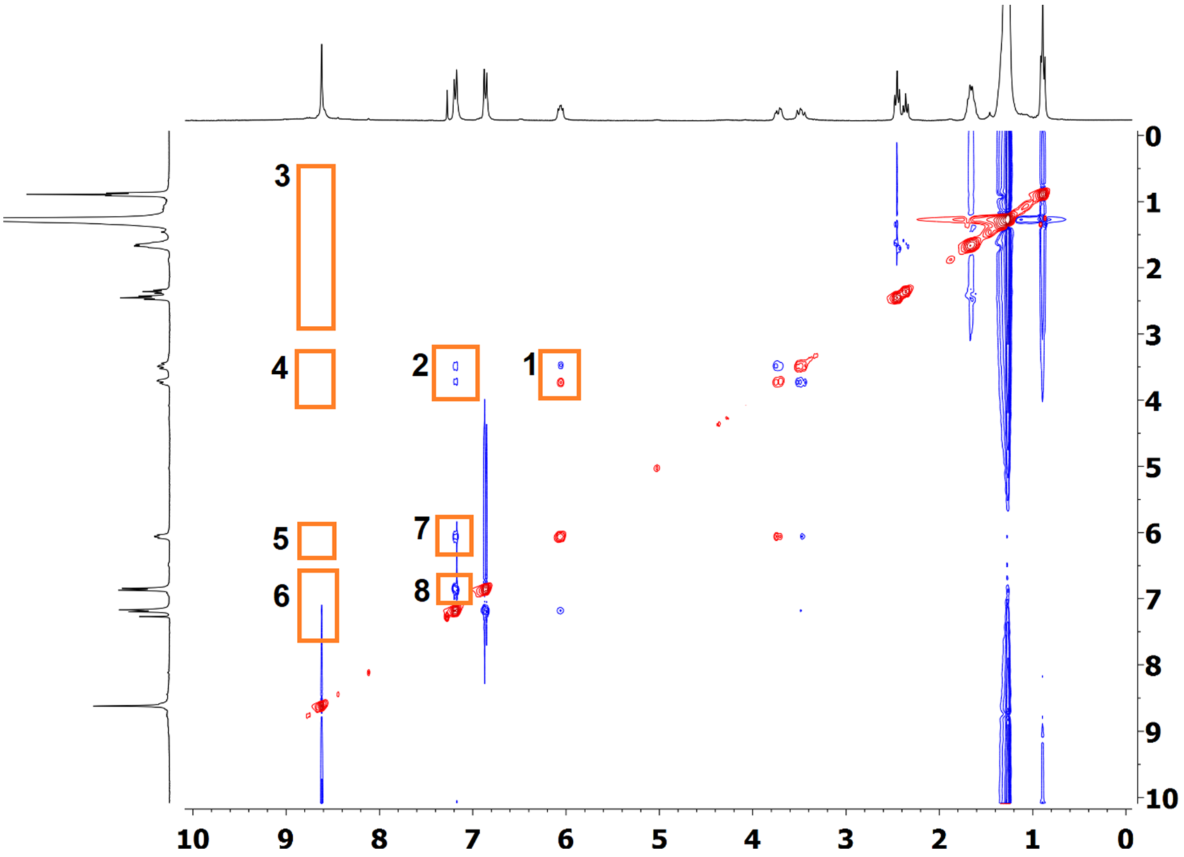

Figure S3. ROESY NMR (300 MHz, 298 K, $CDCl_3$) spectrum of **1**.

Table S2. Assignment of the ROE couplings recorded on the ROESY NMR spectrum of **1**.

Coupling	Assignment
1	Intramolecular coupling between CH and CH_2 groups in the Tyr side chain
2	Intramolecular coupling between CH_2 and Ar–H in the Tyr side chain
7	Intramolecular coupling between CH and Ar–H in the Tyr side chain
8	Intramolecular coupling between Ar–H and Ar–H in the Tyr side chain

Table S3. Important ROE couplings indicative for an *anti* conformation, which have been not recorded on the ROESY NMR spectrum of **1**.

Coupling	Assignment
3	Not recorded: Couplings between NDI core and ester side chain. This coupling would be indicative for the *anti* conformation.[S3]
4	Not recorded: Coupling between NDI core and CH_2 from Tyr side chain
5	Not recorded: Coupling between NDI core and CH from Tyr side chain.
6	Not recorded: Couplings between NDI core and Ar–H from Tyr side chain. This coupling would be indicative for the *anti* conformation.[S3]

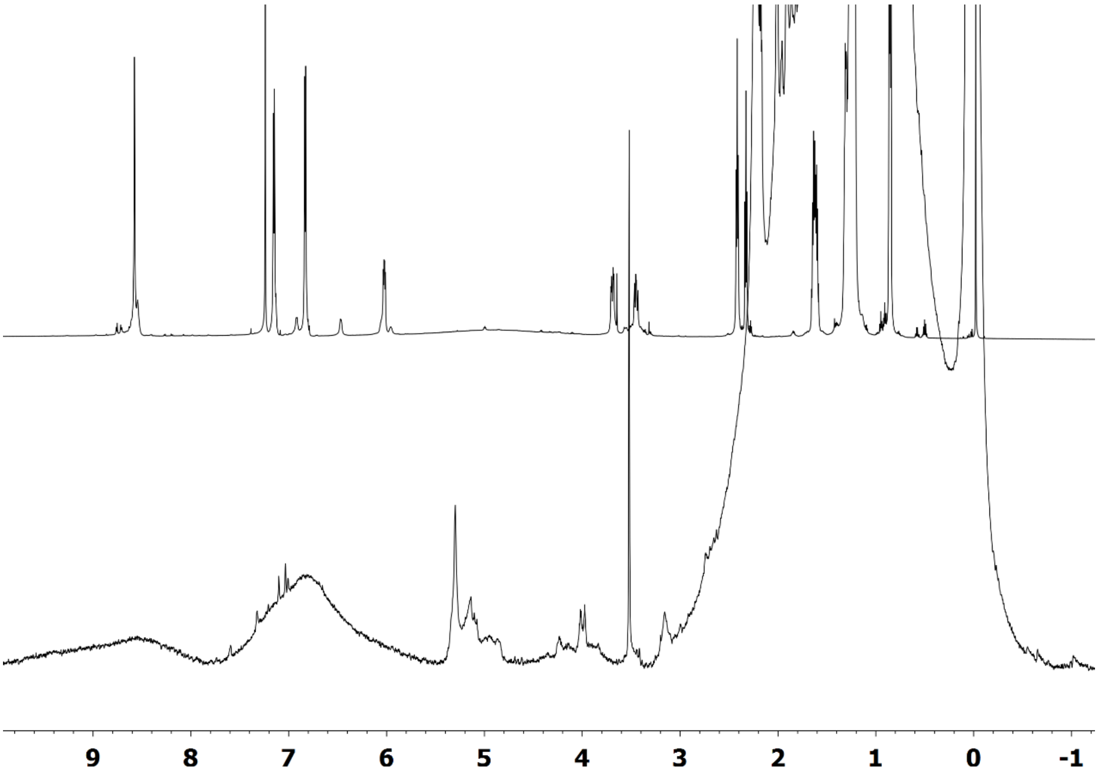

Figure S4. Comparison between ¹H NMR (700 MHz, 298 K) spectra of **1** recorded in $CHCl_3$ (top) and MCH-d_{14} (bottom).

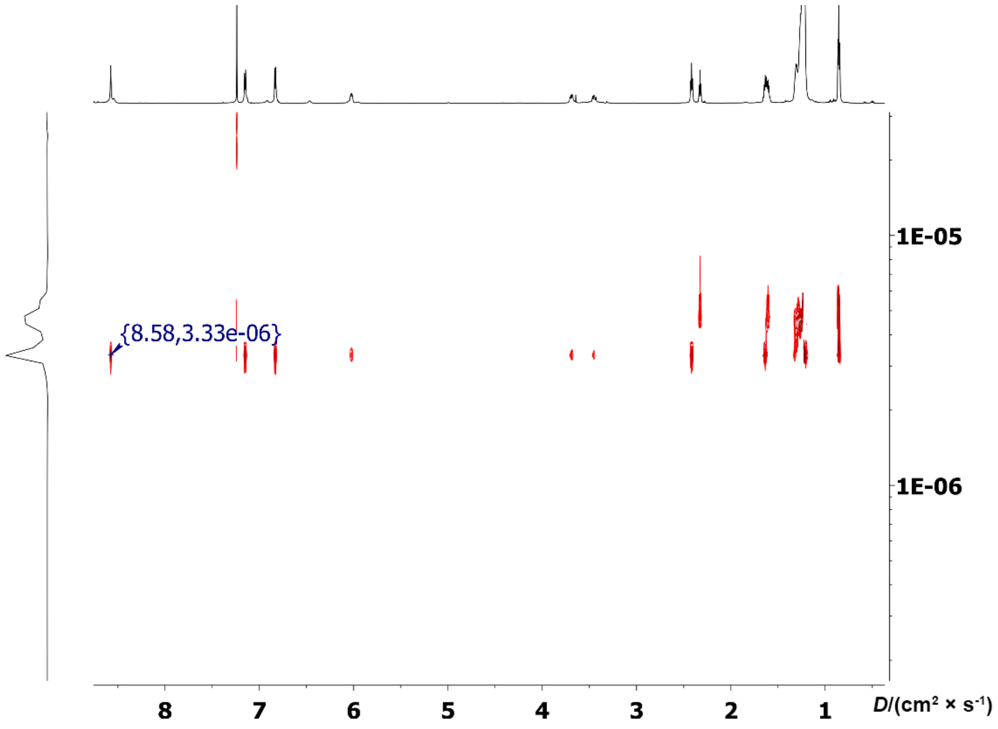

Figure S5. ¹H DOSY NMR (700 MHz, $CDCl_3$, 298 K) spectrum of **1**. A value for the diffusion coefficient of $D = 3.33 \times 10^{-10}$ $m^2 \times s^{-1}$ was found.

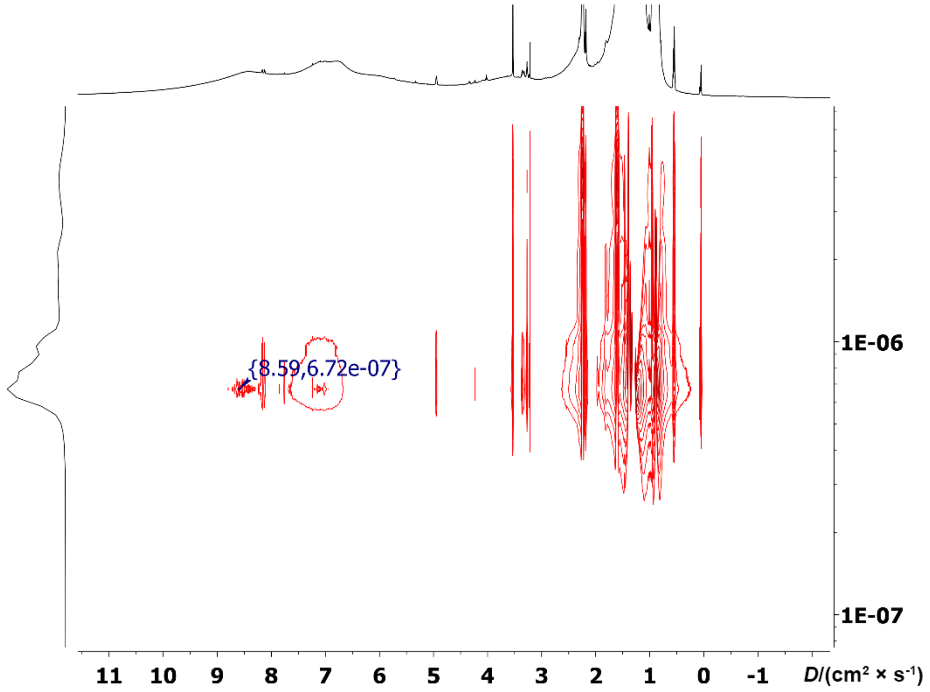

Figure S6. ^{1}H DOSY NMR (700 MHz, MCH-d_{14}, 298 K) spectrum of **1**. A value for the diffusion coefficient of $D = 6.72 \times 10^{-11}$ $m^2 \times s^{-1}$ was found.

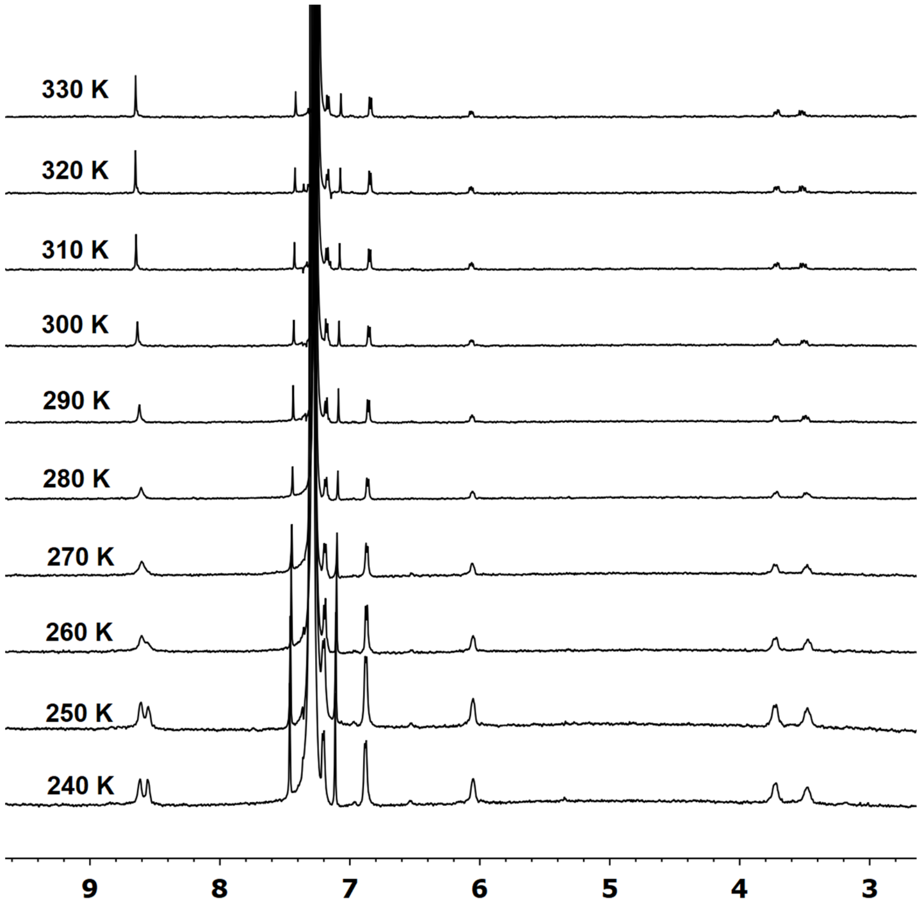

Figure S7. VT ^{1}H NMR (600 MHz, $CDCl_3$) spectra of **1** at C = 1.0×10^{-4} M (cooling). Spectra were referenced to TMS, $\delta = 0.00$ ppm.

5. Supplementary spectra and plots

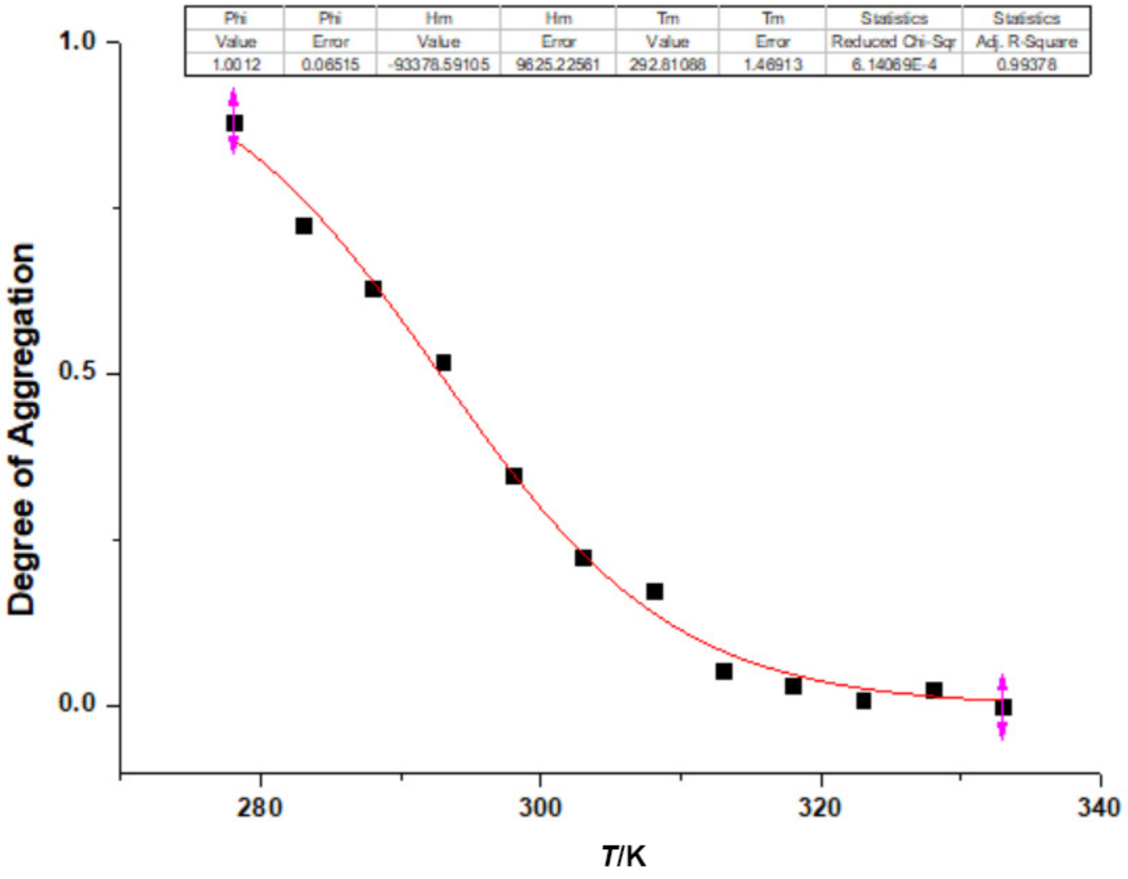

Figure S8. VT CD plot recorded during cooling of **1** solution in $CHCl_3$ at C = 1.0 × 10^{-4} M and λ = 383 nm. Red trace: isodesmic fit. The equation used for fitting was taken from reference.[S1]

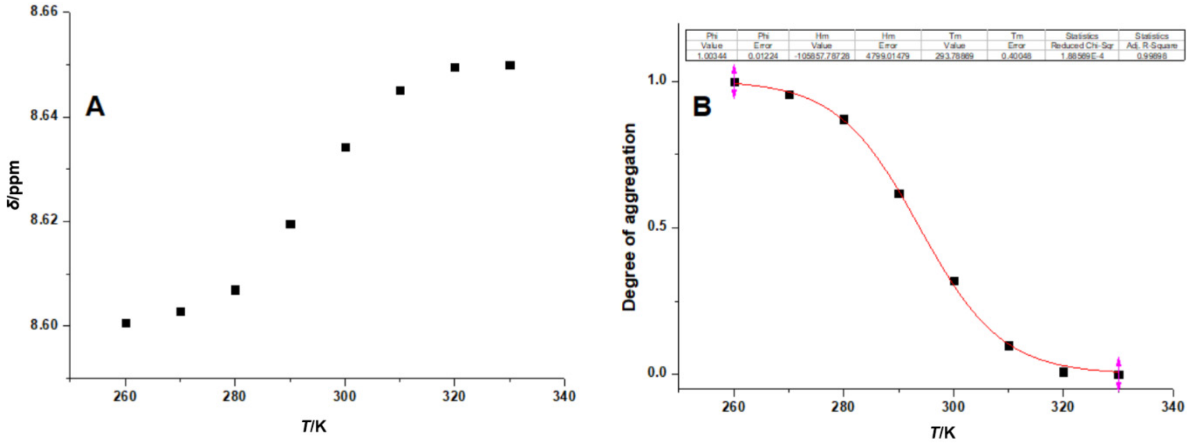

Figure S9. a) VT ^{1}H NMR plot showing the NDI proton resonance as function of temperature (Each spectrum was referenced to TMS, δ = 0.00 ppm) recorded for **1** in $CDCl_3$ at C = 1.0 × 10^{-4} M. b) Isodesmic fit to the normalized VT ^{1}H NMR plot (NDI signal). The equation used for fitting was taken from reference.[S1]

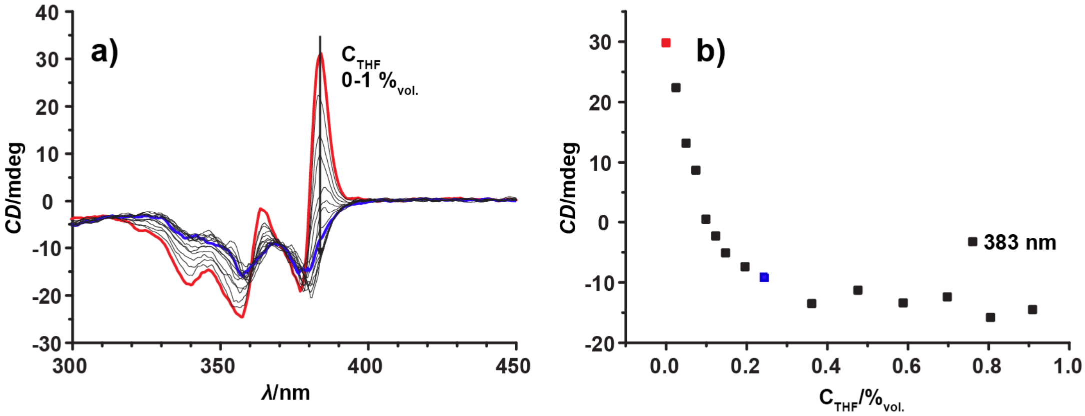

Figure S10. a) CD spectra recorded during the titration of a solution of **1** in $CHCl_3$ with THF (1.0×10^{-4} M, C_{THF} = 0.0–1.0% v/v). Spectra at 0.0% and 0.25% THF are marked in red and blue, respectively. b) Changes in the CD intensity at λ = 383 nm upon titration of a solution of **1** in $CHCl_3$ with THF (1.0×10^{-4} M, C_{THF} = 0.0–1.0% v/v). The colored markers in the graph correspond to the full CD spectra of the same color.

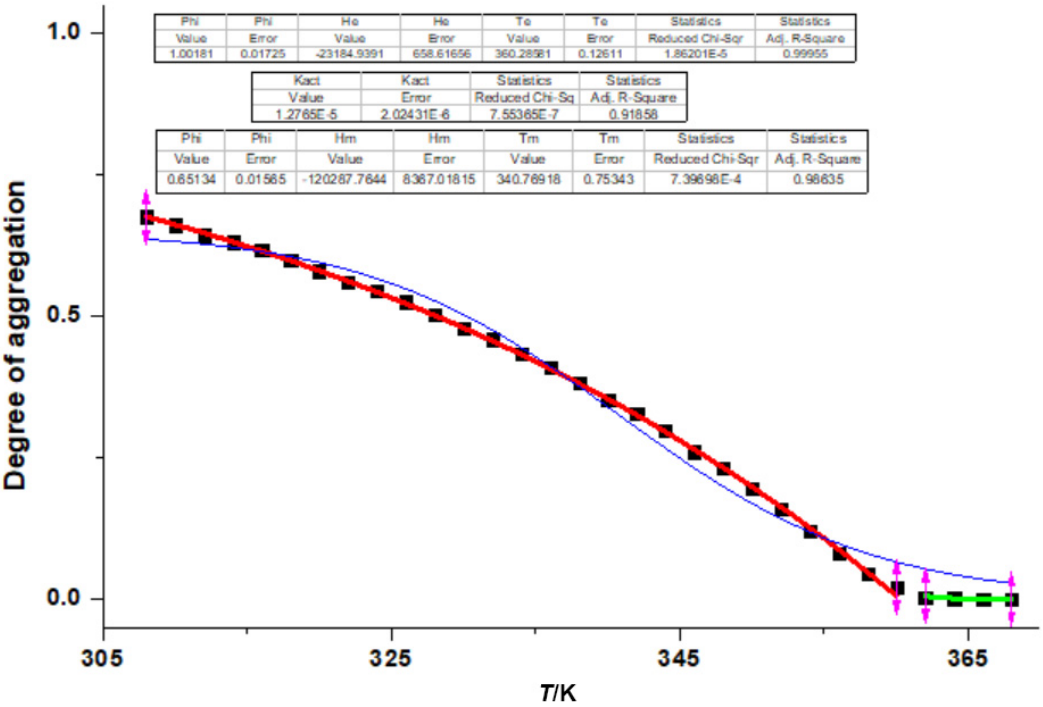

Figure S11. VT UV plot recorded during cooling of **1** solution in MCH at λ = 383 nm (5.0×10^{-5} M, cooling rate: -1 K × min^{-1}). Red trace: elongation fit, green trace: nucleation fit, blue trace: isodesmic fit. The fitting equations were taken from references.[S1,S2] The isodesmic model is clearly unable to describe the experimental data; while the cooperative (nucleation-elongation) model can.

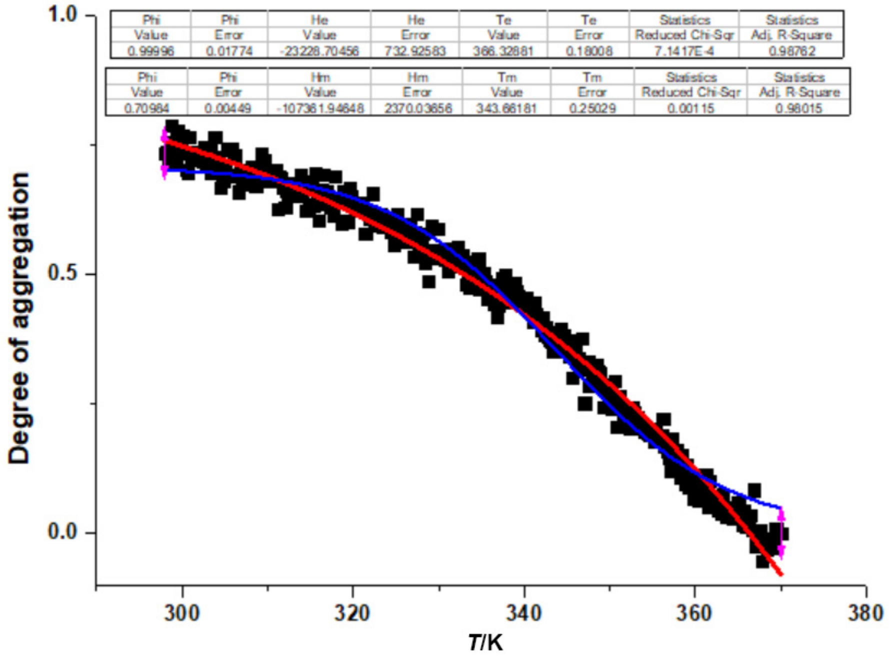

Figure S12. VT CD plot recorded during cooling of **1** solution in MCH (5.0×10^{-5} M, cooling rate: -1 K $\times$ min^{-1}). Red trace: elongation fit, blue trace: isodesmic fit. The fitting equations were taken from references.[S1,S2] From the figure it can be concluded that the cooperative (nucleation-elongation) model can better describe the experimental data than the isodesmic model.

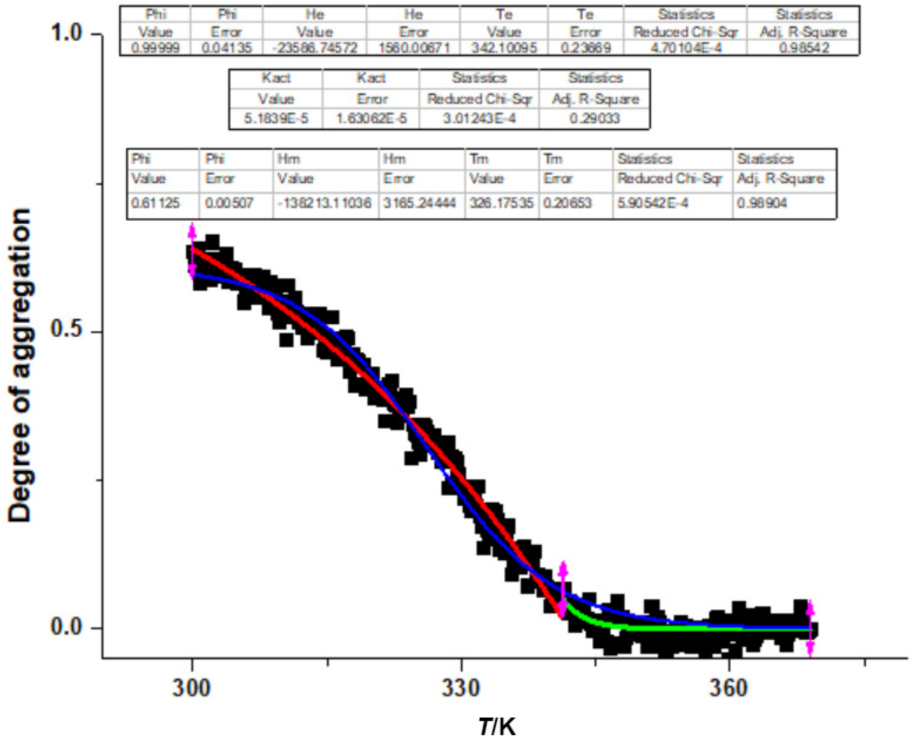

Figure S13. VT CD plot recorded during cooling of **1** solution in MCH (2.5×10^{-5} M, cooling rate: -1 K $\times$ min^{-1}). Red trace: elongation fit, blue trace: isodesmic fit. The fitting equations were taken from references.[S1, S2] From the figure it can be concluded that the cooperative (nucleation-elongation) model can better describe the experimental data than the isodesmic model.

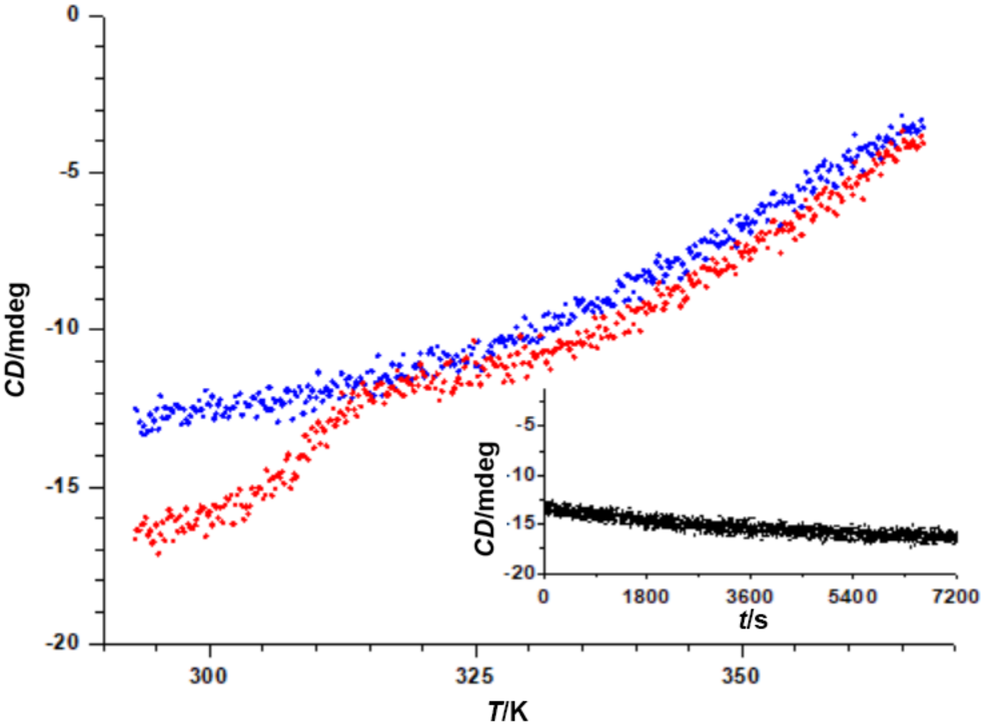

Figure S14. VT CD plot (blue) recorded during cooling of **1** in MCH solution at 5.0 × 10^{-5} M. VT CD plot (red) recorded during reheating of this solution after 2 h at 293 K (heating/cooling rate: ±1 K × min^{-1}). Inset: Time-dependent CD intensity monitored at 293 K. All data points were recorded at λ = 386 nm.

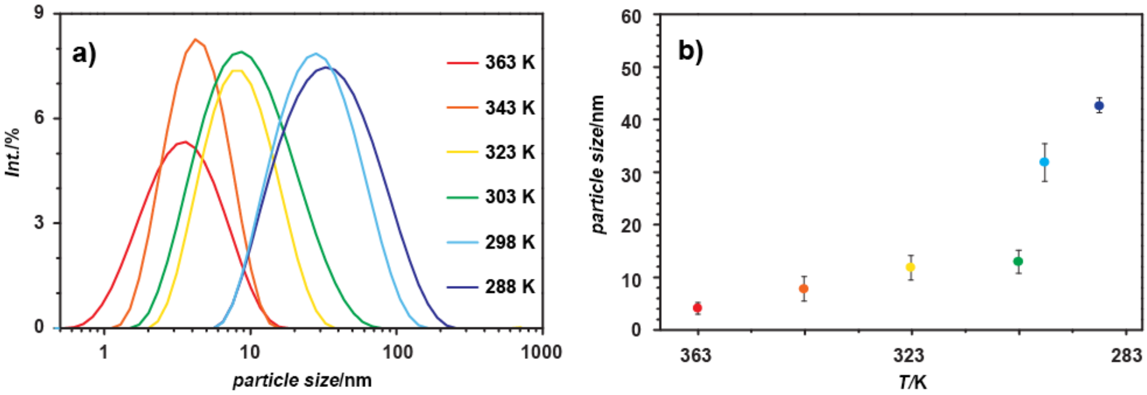

Figure S15. a) VT DLS plots recorded during cooling of **1** in MCH solution at 1.0 × 10^{-3} M. (equilibration time t_{eq} = 30 min). b) Average particle size as a function of temperature plot, as obtained during cooling of a solution of **1** in MCH (1.0 × 10^{-3} M, T = 363–288 K, equilibration time t_{eq} = 30 min) The average value and standard deviation from 6 experiments are shown.

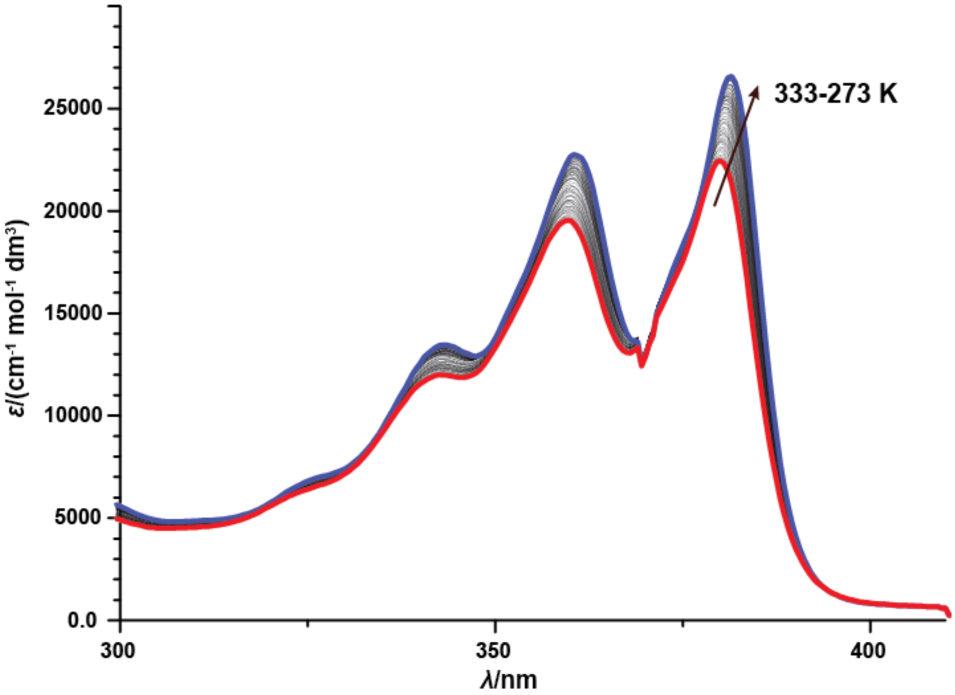

Figure S16. VT-UV spectra recorded during cooling of a solution of **1** in $CHCl_3$ at 1.0×10^{-4} M.

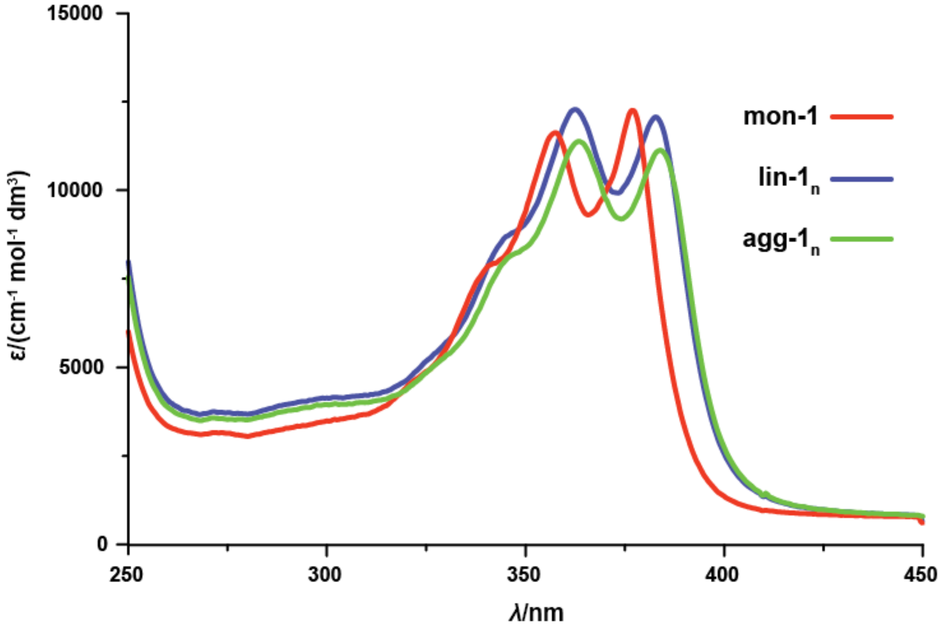

Figure S17. UV spectra of **mon-1**, **lin-1$_n$** and **agg-1$_n$** in MCH solution at 5.0×10^{-5} M. Spectra were recorded as follows: **mon-1** stable spectrum at 370 K, **lin-1$_n$** spectrum at 308 K after cooling from 370 K with the cooling rate: -1 K × min^{-1}, **agg-1$_n$** stable spectrum after 24 h at 288 K.

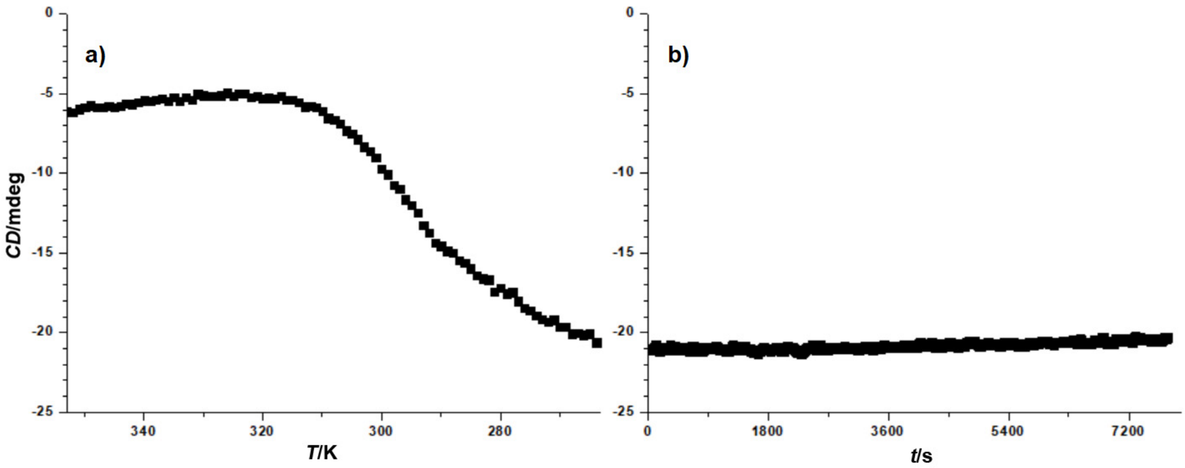

Figure S18. a) VT CD plot recorded during cooling of **1** in MCH/$CHCl_3$ 75/25 solution. (λ = 388 nm, 1.0×10^{-4} M, T = 353–263 K, cooling rate –1 K × min^{-1}) b) Time-dependent evolution of the CD intensity at 263 K (λ = 388 nm) after cooling a sample from 353 K.

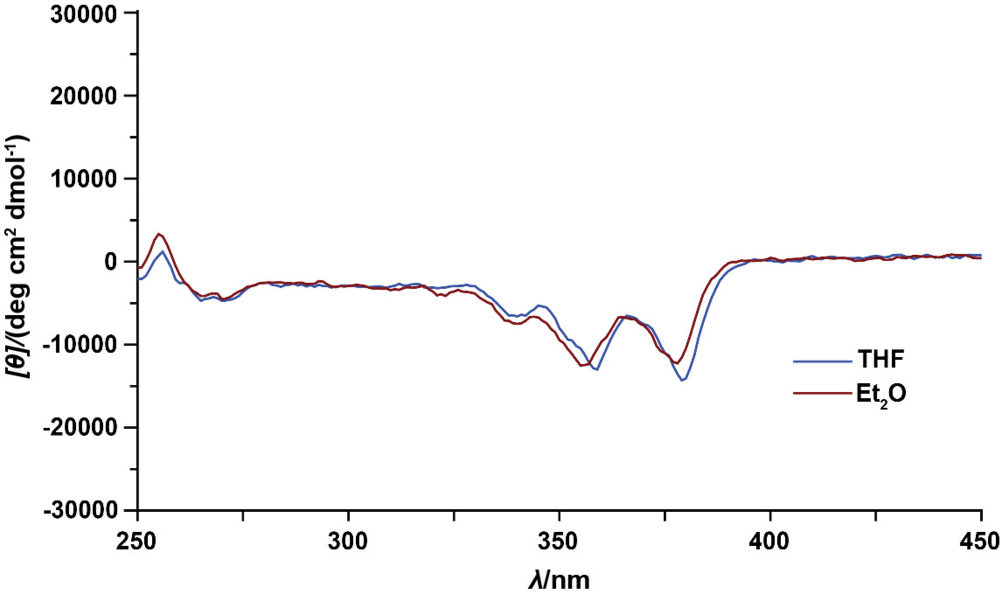

Figure S19. CD spectra of **1** recorded in Et_2O and THF (1.0×10^{-4} M).

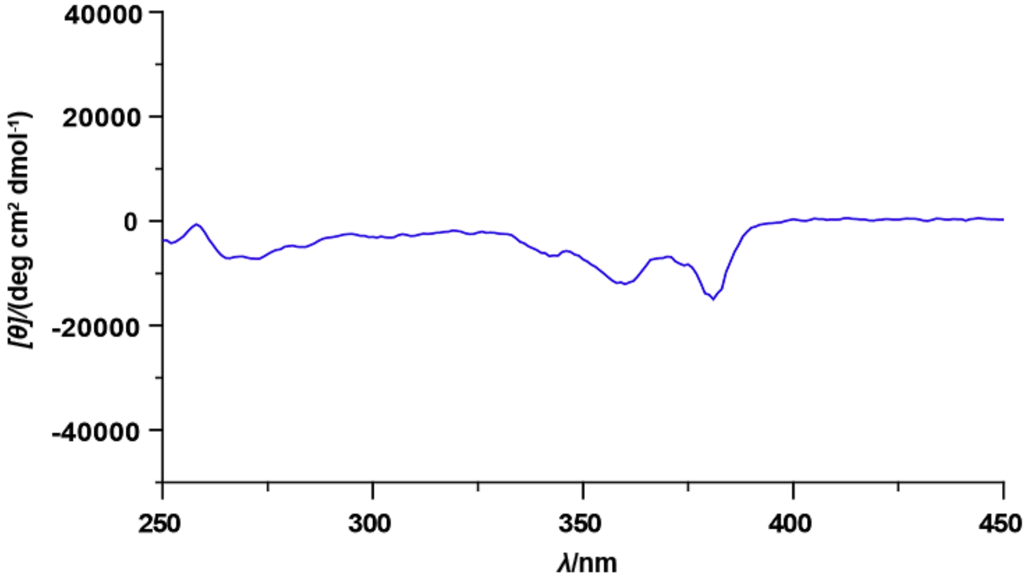

Figure S20. CD spectrum of **1-COO*t*-Bu** recorded in $CHCl_3$ (1.0×10^{-4} M).

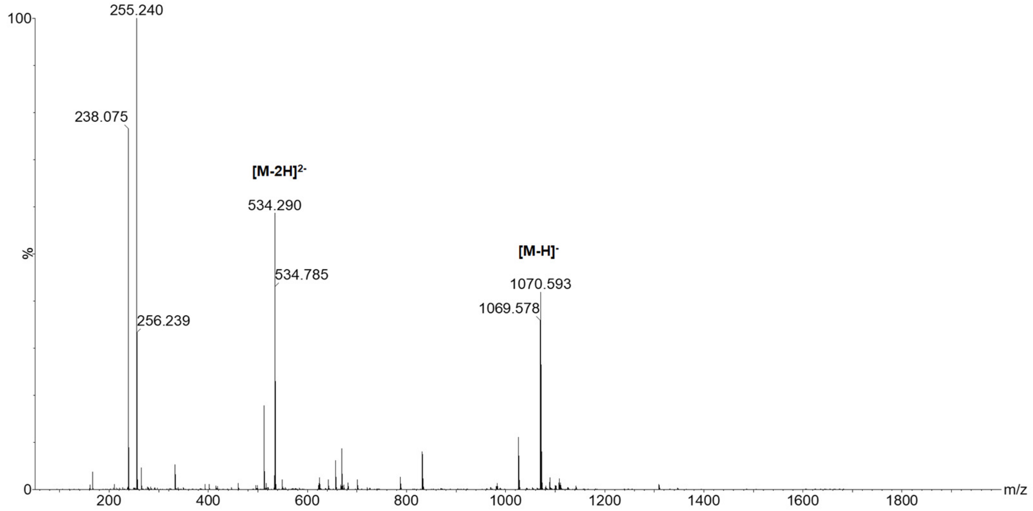

Figure S21. ESI-ToF-MS spectrum of **1** in MeCN/MeOH solution (negative ion mode).

6. Atomic Force Microscopy

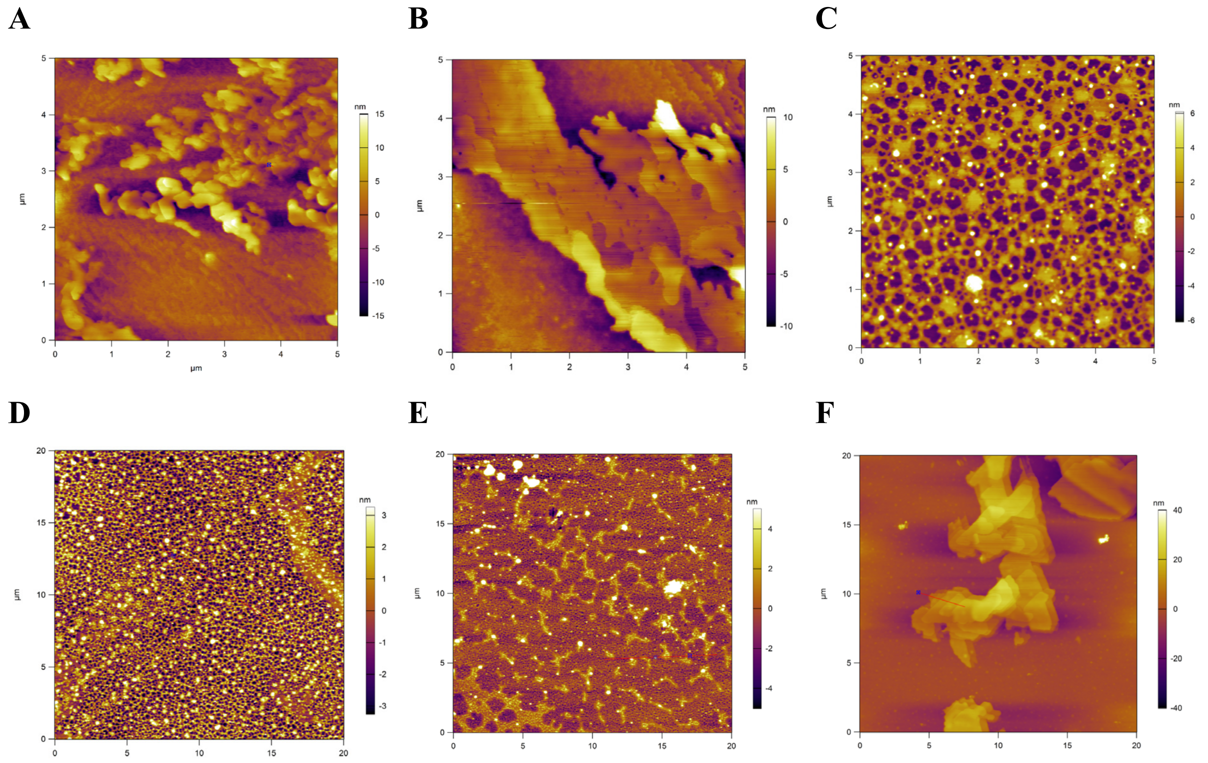

Figure S22. Exemplary AFM images (height profiles) of **1** recorded by drop-casting a solution of **1** in $CHCl_3$ or MCH. A) 5×5 µm profile obtained from MCH solution (1.0×10^{-4} M) deposited on pure SiO_2 substrate. B) 5×5 µm profile obtained from MCH solution (5.0×10^{-5} M) deposited on pure SiO_2 substrate. C) 5×5 µm profile obtained from MCH solution (5.0×10^{-5} M) deposited on hydrophobized (-$SiMe_3$) SiO_2 substrate. D) 20×20 µm profile obtained from MCH solution (5.0×10^{-5} M) deposited on hydrophobized (-$SiMe_3$) SiO_2 substrate. E) 20×20 µm profile obtained from the warm MCH solution (313 K, $5.0{\times}10^{-5}$ M) deposited on hydrophobized (-$SiMe_3$) SiO_2 substrate. F) 20×20 µm profile obtained from $CHCl_3$ solution ($1.0{\times}10^{-4}$ M) deposited on hydrophobized (-$SiMe_3$) SiO_2 substrate.

7. FT-IR

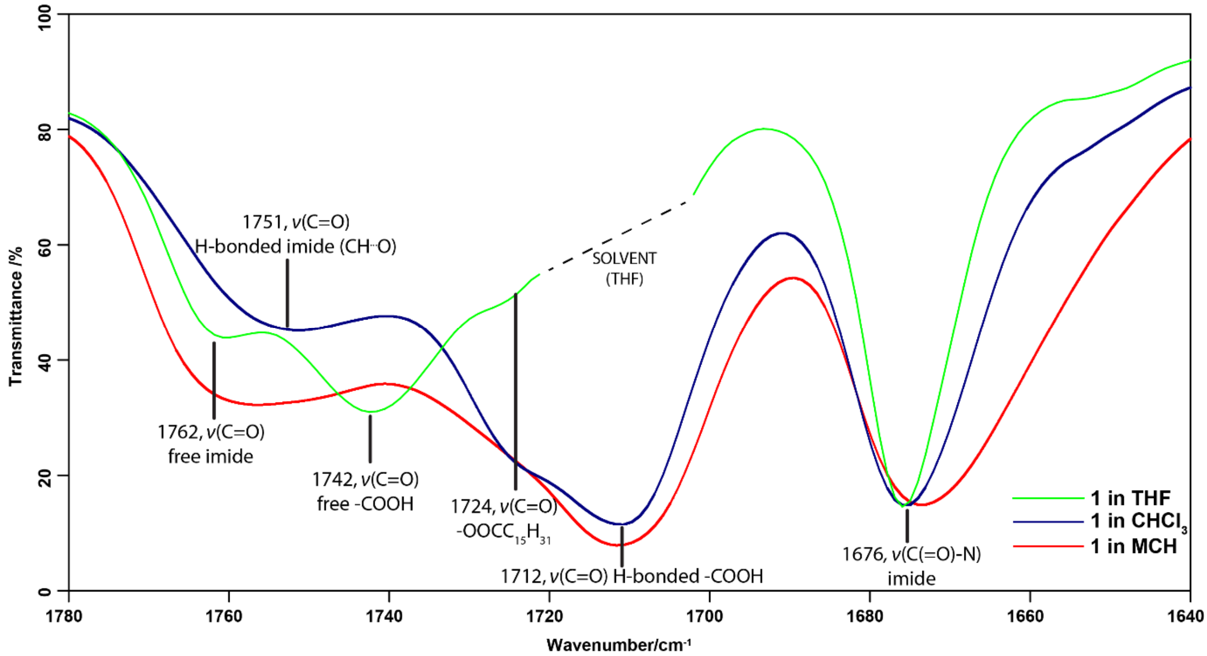

Figure S23. Stacked FT-IR spectra ($\nu_{C=O}$ range) of **1** in THF (green), $CHCl_3$ (blue), and MCH (red) recorded at 3.0×10^{-2} M and 298 K. Full spectra are shown in Figure S24-S26.

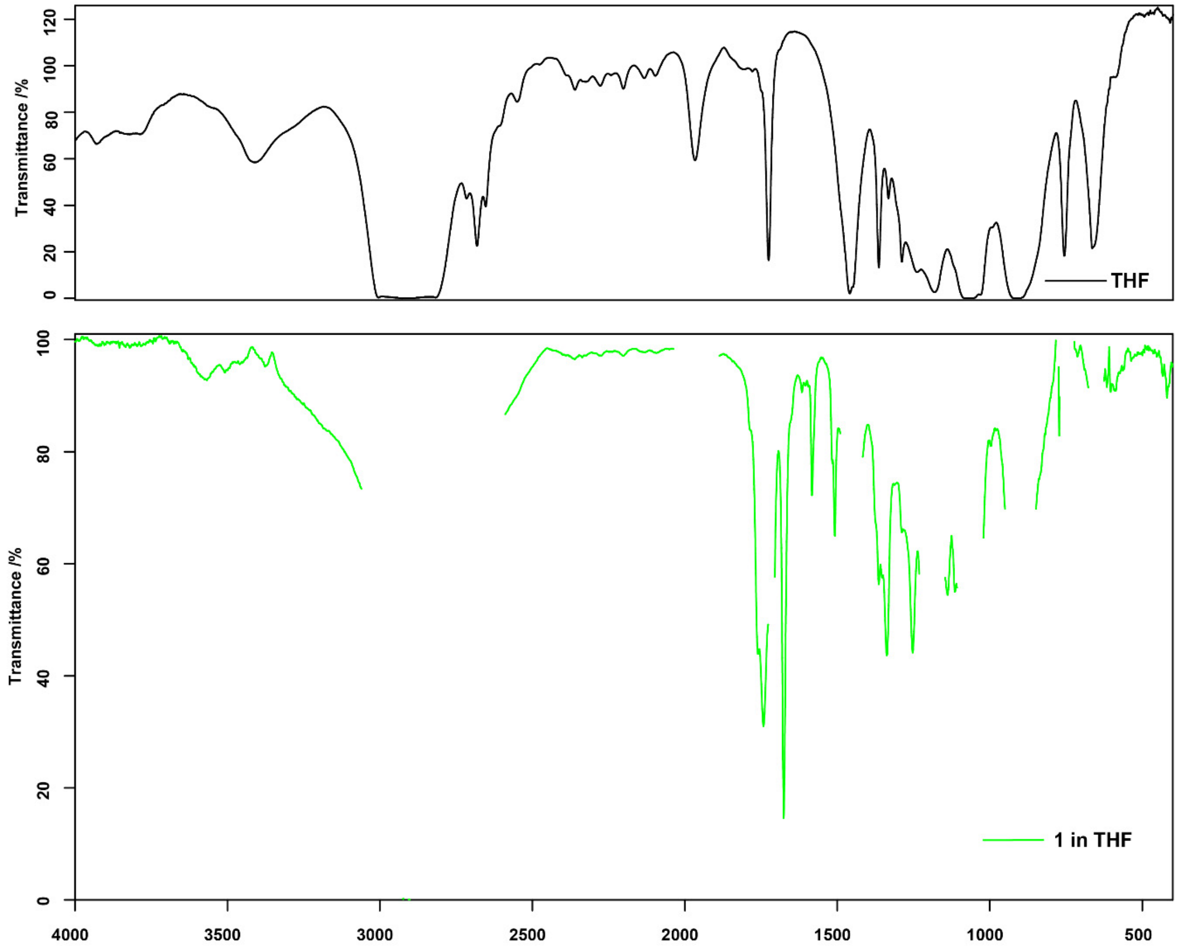

Figure S24. (Solvent-corrected) FT-IR spectrum of **1** in THF recorded at 3.0×10^{-2} M and 298 K. For reference the transmittance of the solvent (THF) is also shown on top (in black).

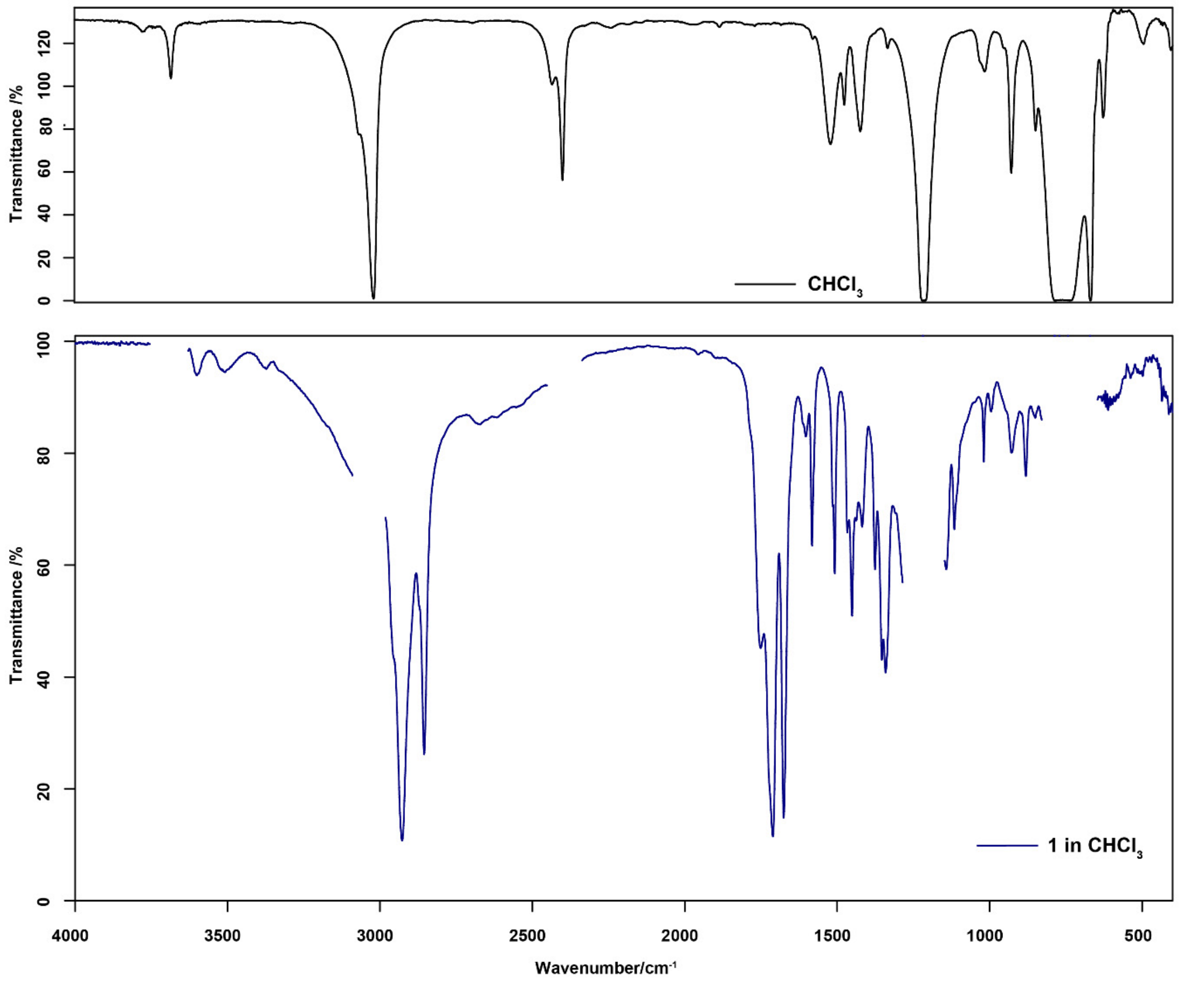

Figure S25. (Solvent-corrected) FT-IR spectrum of **1** in $CHCl_3$ recorded at 3.0×10^{-2} M and 298 K. For reference the transmittance of the solvent ($CHCl_3$) is also shown on top (in black).

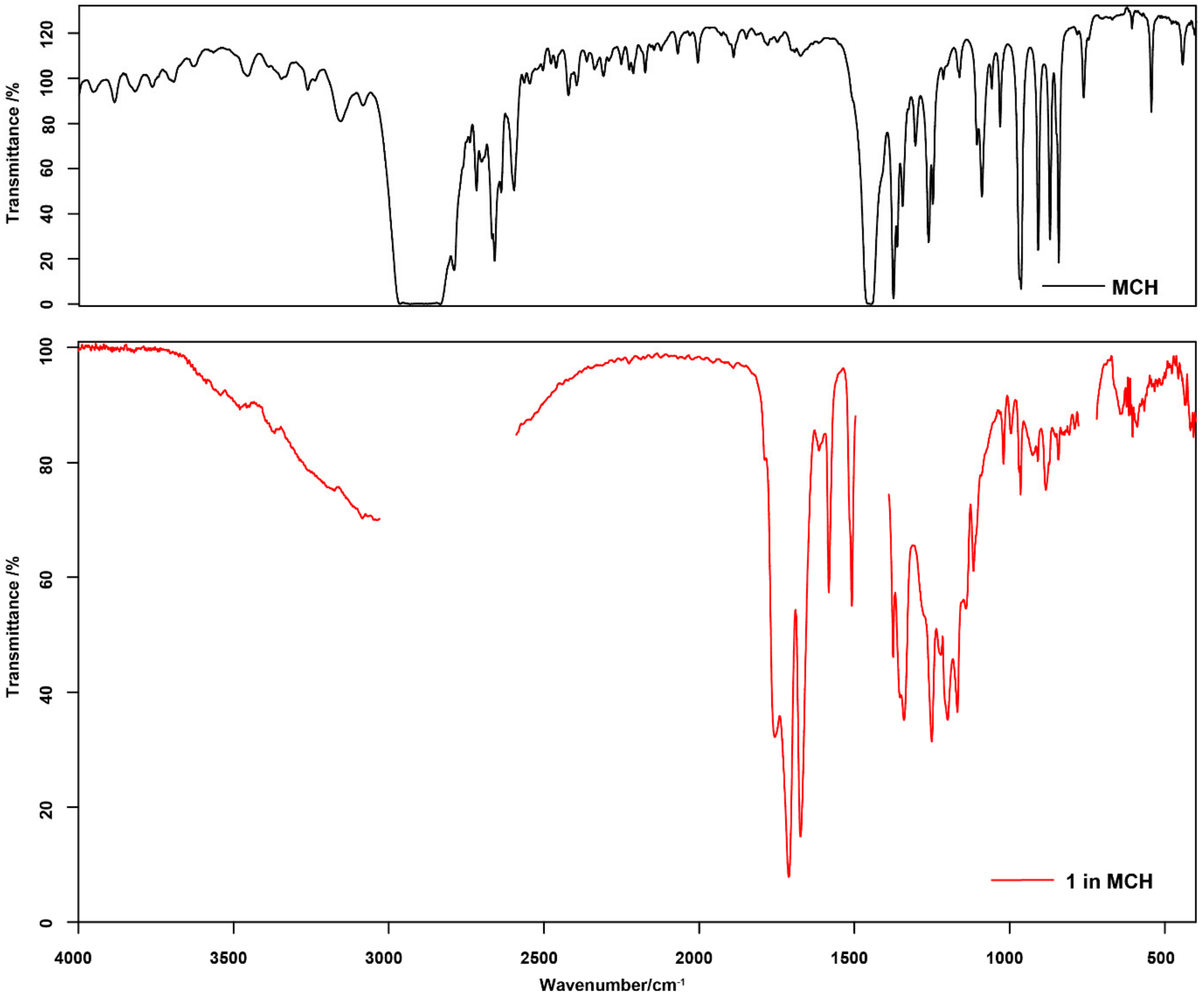

Figure S26. (Solvent-corrected) FT-IR spectrum of **1** in MCH recorded at 3.0×10^{-2} M and 298 K. For reference the transmittance of the solvent (MCH) is also shown on top (in black).

8. References

[S1] M. M. J. Smulders, M. M. L. Nieuwenhuizen, T. F. A. de Greef, P. van der Schoot, A. P. H. J. Schenning, E. W. Meijer, *Chem. Eur. J.* **2010**, *16*, 362.

[S2] M. M. J. Smulders, A. P. H. J. Schenning, E. W. Meijer, *J. Am. Chem. Soc.* **2008**, *130*, 606.

[S3] M. A.Squillaci, G. Markiewicz, A.Walczak, A. Ciesielski, A. R. Stefankiewicz, P. Samorì, *Chem. Commun.* **2017,** *53*, 9713.
